# Understanding the Significance of Hypoxia-Inducible Factors (HIFs) in Glioblastoma: A Systematic Review

**DOI:** 10.3390/cancers16112089

**Published:** 2024-05-30

**Authors:** Emir Begagić, Hakija Bečulić, Amina Džidić-Krivić, Samra Kadić Vukas, Semir Hadžić, Alma Mekić-Abazović, Sabina Šegalo, Emsel Papić, Emmanuel Muchai Echengi, Ragib Pugonja, Tarik Kasapović, Dalila Kavgić, Adem Nuhović, Fatima Juković-Bihorac, Slaviša Đuričić, Mirza Pojskić

**Affiliations:** 1 Department of General Medicine, School of Medicine, University of Zenica, 72000 Zenica, Bosnia and Herzegovina; 2 Department of Neurosurgery, Cantonal Hospital Zenica, 72000 Zenica, Bosnia and Herzegovina; hakija.beculic@dl.unze.ba; 3 Department of Anatomy, School of Medicine, University of Zenica, 72000 Zenica, Bosnia and Herzegovina; 4 Department of Neurology, Cantonal Hospital Zenica, 72000 Zenica, Bosnia and Herzegovinasamrakadic@gmail.com (S.K.V.); 5 Department of Physiology, Faculty of Medicine, University of Tuzla, 75000 Tuzla, Bosnia and Herzegovina; 6 Department of Oncology, Cantonal Hospital Zenica, 72000 Zenica, Bosnia and Herzegovina; 7 Department of Laboratory Technologies, Faculty of Health Studies, University of Sarajevo, 71000 Sarajevo, Bosnia and Herzegovina; sabina.segalo@fzs.unsa.ba (S.Š.); e.papic99@gmail.com (E.P.); 8 College of Health Sciences, School of Medicine, Kenyatta University, Nairobi 43844-00100, Kenya; 9 Department of General Medicine, School of Medicine, University of Sarajevo, 71000 Sarajevo, Bosnia and Herzegovina; ademnuhovic@gmail.com; 10 Department of Pathology, Cantonal Hospital Zenica, 72000 Zenica, Bosnia and Herzegovina; 11 Department of Pathology, School of Medicine, University of Zenica, 72000 Zenica, Bosnia and Herzegovina; slavisa.djuricic@gmail.com; 12 Department of Neurosurgery, University Hospital Marburg, 35033 Marburg, Germany

**Keywords:** hypoxia, brain neoplasms, glial tumors, microenvironment, targeted therapy

## Abstract

**Simple Summary:**

This study explores hypoxia-inducible factors (HIFs) in glioblastoma development, progression, and treatment. Reviewing 104 relevant studies, it highlights diverse global contributions, with China leading at 23.1%. The most productive year was 2019, contributing 11.5% of the studies. Key factors studied included HIF1α, HIF2α, osteopontin, and cavolin-1, involving pathways such as GLUT1, GLUT3, VEGF, PI3K-Akt-mTOR, and ROS. HIF expression correlates with glioblastoma progression, survival, neovascularization, glucose metabolism, migration, and invasion. Overcoming treatment resistance and the lack of biomarkers is crucial for integrating HIF-related therapies into glioblastoma treatment to improve patient outcomes.

**Abstract:**

Background: The study aims to investigate the role of hypoxia-inducible factors (HIFs) in the development, progression, and therapeutic potential of glioblastomas. Methodology: The study, following PRISMA guidelines, systematically examined hypoxia and HIFs in glioblastoma using MEDLINE (PubMed), Web of Science, and Scopus. A total of 104 relevant studies underwent data extraction. Results: Among the 104 studies, global contributions were diverse, with China leading at 23.1%. The most productive year was 2019, accounting for 11.5%. Hypoxia-inducible factor 1 alpha (HIF1α) was frequently studied, followed by hypoxia-inducible factor 2 alpha (HIF2α), osteopontin, and cavolin-1. Commonly associated factors and pathways include glucose transporter 1 (GLUT1) and glucose transporter 3 (GLUT3) receptors, vascular endothelial growth factor (VEGF), phosphoinositide 3-kinase (PI3K)-Akt-mechanistic target of rapamycin (mTOR) pathway, and reactive oxygen species (ROS). HIF expression correlates with various glioblastoma hallmarks, including progression, survival, neovascularization, glucose metabolism, migration, and invasion. Conclusion: Overcoming challenges such as treatment resistance and the absence of biomarkers is critical for the effective integration of HIF-related therapies into the treatment of glioblastoma with the aim of optimizing patient outcomes.

## 1. Introduction

Glioblastoma is a highly aggressive grade 4 glioma with an annual incidence of approximately six cases per 100,000 persons in older adults and a 15–20% proportion of all brain tumors in pediatric patients. In children, glioblastoma is highly invasive and leads to an 80% recurrence rate within two years of treatment. Survival rates are dismal, with less than 2% of the adults surviving more than three years after diagnosis [1,2,3,4].

Recent advances favor molecular analysis for the prognosis of glioblastoma, especially in younger patients where molecular factors are more important than histological grading. Biomarkers such as isocitrate dehydrogenase (IDH) mutations and O6-methylguanine DNA methyltransferase (MGMT) methylation status support prognosis [5]. However, the final diagnosis of glioblastoma depends on the surgical biopsy, which is crucial for the detection of hypoxic tumor niches manifested by vascular proliferation and tissue necrosis. Hypoxia, which is prevalent in solid tumors such as glioblastoma, is due to reduced oxygen levels, which are particularly dangerous in the oxygen-dependent brain [6]. Tumor progression exacerbates hypoxia and leads to uncontrolled neovascularization that perpetuates the cycle of inadequate oxygen supply. Hypoxia-induced angiogenesis is typical of the progression that occurs in escalation-grade astrocytomas and is characterized by central necrosis and pseudo-palisades on magnetic resonance imaging (MRI) scans [7].

Hypoxia-inducible factor (HIF) emerges as a key molecule in promoting neovascularization in hypoxic niches, which is critical for tumor progression [8]. To date, the involvement of the hypoxic microenvironment in carcinogenesis has been extensively validated across various tumor types [9], particularly in pancreatic cancer, wherein hypoxic conditions have been shown to facilitate metastasis and drug resistance [10]. The HIF1α and HIF-1β subunits form an active heterodimer that initiates the transcription of over 40 hypoxia-responsive genes, including erythropoietin (EPO), insulin-like growth factor 2 (IGF2), vascular endothelial growth factor (VEGF), and angiopoietin (Ang)-1 and -2 [11]. HIF also upregulates platelet-derived growth factor (PDGF) proteins and activates oncogenic signaling pathways such as MAPK/RAS and PI3K/AKT [9]. HIF1α responds acutely to hypoxia, while HIF2α regulates tumor cell response to chronic hypoxia, making it a potential therapeutic target. BEV targeting VEGF-A shows promise in inhibiting HIF1α, especially in patients with chemoresistance [12,13]. However, the histologic and molecular heterogeneity of glioblastoma poses a challenge and requires research into novel multimodal therapies targeting hypoxia and HIF signaling pathways, including immunotherapy and nanoscale drug delivery [12,13]. Therefore, the aim of this systematic review is to investigate the role of hypoxia and HIFs in the development, progression, and therapeutic potential of glioblastoma.

## 2. Materials and Methods

### 2.1. Study Design and Registration

A systematic review of the literature was conducted to investigate the role of hypoxia and HIFs in glioblastoma. The methodology followed the established PRISMA (Preferred Reporting Items for Systematic Reviews and Meta-Analyses) guidelines [14]. This systematic review has been registered in the Open Science Framework (OSF) Register with the unique identifier OSF-REGISTRATIONS-8GD9K-V1 [15].

### 2.2. Search Strategy

On 15 January 2024, a search was conducted using the PICOS method to define the main search terms (Table 1). Three databases were searched: MEDLINE (PubMed), Web of Science (Clarivate Analytics, Philadelphia, PA, USA), and Scopus. The keywords “glioblastoma” and “hypoxia-inducible factors” were searched. A detailed search strategy can be found in Appendix A. Following the PRISMA guidelines, a checklist can be found in Appendix B.

### 2.3. Study Selection

#### 2.3.1. Inclusion and Exclusion Criteria

Strict inclusion and exclusion criteria were applied when conducting this systematic review to ensure the selection of relevant articles that contribute significantly to the understanding of the role of HIFs in glioblastoma. The inclusion criteria focused on articles that were written in English, directly related to the interaction between HIFs and glioblastoma, and contained data relevant to the objectives of the study. Conversely, the exclusion criteria were carefully defined to refine the selection process and exclude articles that may not align with the aims of the study. Excluded articles included book chapters, conference papers, reviews, non-English language literature, and articles that did not contain relevant data.

#### 2.3.2. Included Studies

**Figure 1:**
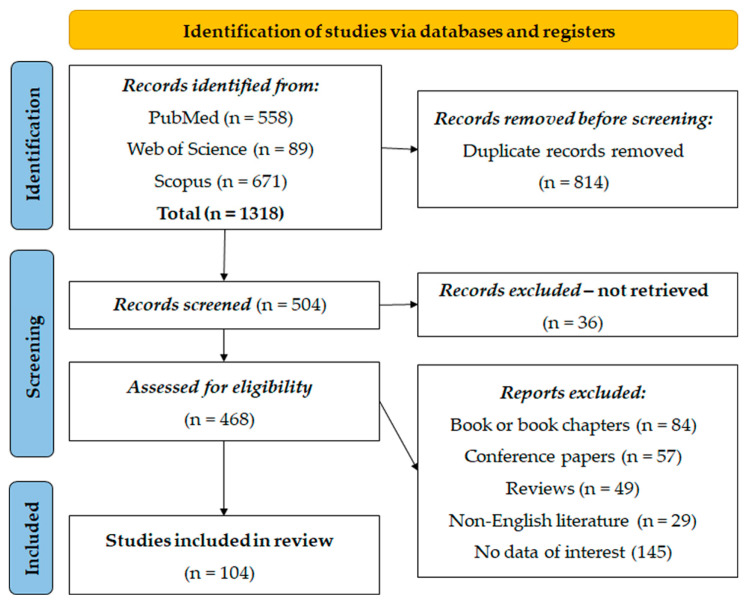
PRISMA flow diagram.

A total of 1318 entries were identified from PubMed (n = 558), Web of Science (n = 89), and Scopus (n = 671). Prior to the screening, 814 duplicate entries were removed using the EndNote software (21.3) for referencing. After automatic deduplication, all the remaining duplicate manuscripts were manually excluded. After this first step, 504 records were screened, and 36 records were excluded because they could not be found. Subsequently, 468 records were screened for eligibility, resulting in the exclusion of 84 book or book chapters, 57 conference papers, 49 reviews, 29 articles from non-English literature, and 145 articles without relevant data. Finally, 104 studies were deemed suitable and included in the systematic re-examination for analysis (Figure 1).

### 2.4. Data Extraction

Data extraction from the studies was performed for didactic purposes, whereby the studies were divided into laboratory and clinical studies based on the tracking of different variables. The laboratory studies were further divided into genetic studies and drug-related studies as well as combined studies. For genetic studies, variables such as authors, country (year), study design, species, cell line(s), targeted HIF, related factors, role of HIF and related factors, gene modification, and the effect of gene modification were tracked. For drug studies, variables such as reference, country (year), study design, species, cell line(s), targeted HIF, related factors, role of HIF and related factors, target/system therapy, and pharmacologic effects were monitored. Combined laboratory studies tracked similar variables along with targeted therapy and pharmacologic effects. Clinical studies were reviewed for variables such as authors, country (year), study design, sample size (N), age, gender distribution, targeted HIF(s), and outcomes. The studies were first extracted into a single file using the EndNote software and then deduplicated. Data extraction was performed by eight researchers under the supervision of three senior researchers. Ambiguities in data extraction were resolved through online meetings and a final consensus.

### 2.5. Statistical Analysis

Descriptive statistics were used to present frequencies and absolute numbers, providing a quantitative summary of various key factors associated with the use of HIFs in glioblastoma. To improve the clarity and interpretation of results, graphical visualization was performed using Microsoft Excel (version 2021, Microsoft Corporation, Washington, DC, USA). BioRender (https://www.biorender.com/, accessed on 10 April 2024) license number RY26P9F0AG was used to design the scientific illustrations in the manuscript.

## 3. Results

### 3.1. Included Studies’ Characteristics

**Figure 2:**
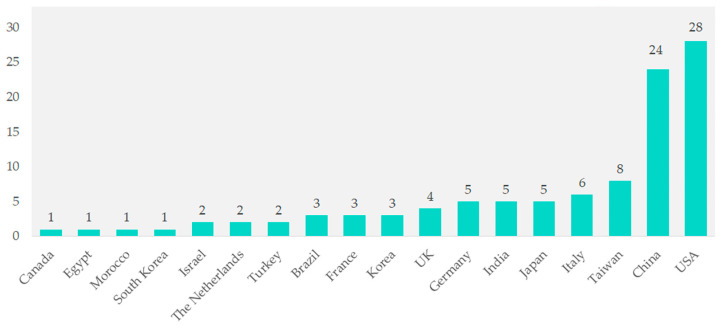
Geographical distribution of included studies.

Among the 104 studies, contributions came from various countries. Studies from Canada, Egypt, Morocco, South Korea, Israel, the Netherlands, and Turkey together made up 1% of the total. Brazil, France, and Korea each contributed three studies (2.9%). The UK contributed four studies (3.8%), while Germany, India, and Japan each contributed five studies (4.8%). Italy contributed six studies (5.8%), and Taiwan contributed eight studies (7.7%). Significant contributions came from China with 24 studies (23.1%) and the United States with 28 studies (26.9%) (Figure 2).

**Figure 3:**
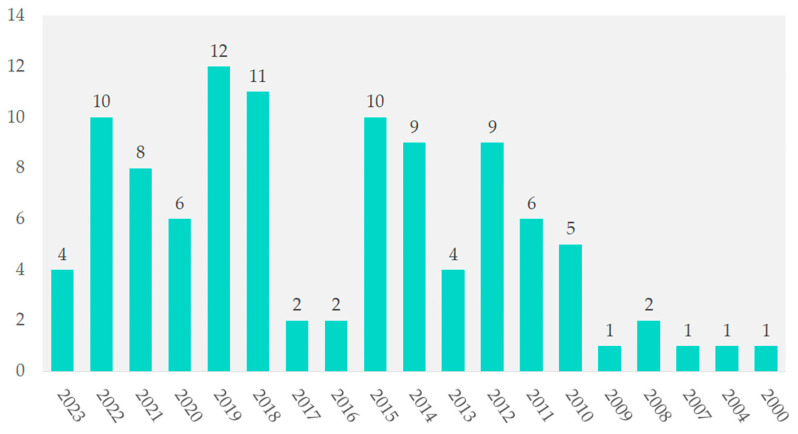
Temporal distribution of included studies.

The years 2000, 2004, 2007, and 2009 each contributed 1%. In contrast, 2019 had 12 studies (11.5%), followed by 2018 with 11 studies (10.6%). From 2012 to 2022, annual contributions ranged from 8.7% to 9.6%. In 2021, the contribution was 7.7%, as shown in Figure 3.

**Figure 4:**
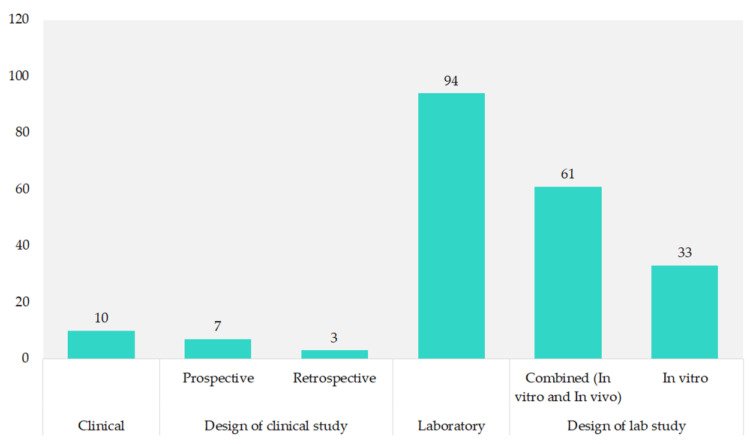
Study design of included studies.

Most of the studies (90.4%) were laboratory-based, with 58.7% combining in vitro and in vivo methods. Pure in vitro studies made up 31.7%. Clinical research was less common, comprising 9.6% of the total, with prospective studies at 6.7% and retrospective studies at 2.9%, as shown in Figure 4.

### 3.2. Role of HIF-Related Gene Modification in the Treatment of Glioblastoma 

Table 2 shows 48 of the 94 laboratory studies (51%) that investigated the role of HIFs using different genetic methods in animal species and glioblastoma models. All the studies investigated HIF1α, while four studies also investigated the role of HIF2α in addition to HIF1α [14,15,16,17]. Among the included studies, deletion, overexpression, and transduction each accounted for 2.08% of the total (N = 1). Combined techniques and knockout methods each accounted for 6.25% (N = 3), while transfection was used in 22.92% of the studies (N = 11). Knockdown techniques in particular accounted for the majority of the research studies (58.33%, N = 28).

#### 3.2.1. HIF’s Mechanisms Explored in Genetic Studies

The included studies have shed light on the complicated mechanisms in which HIF1α is involved. For example, Hashimoto et al. [16] have shown that AMPK boosts ATM expression via the transcription factor Sp1 under severe hypoxia, contributing to radioresistance. Conversely, Ho et al. [17] elucidated the role of MIR210HG in hypoxia-mediated glioma invasion and stemness formation, which is regulated by OCT1 and affects the expression of IGFBP2 and FGFR1. In addition, Ishikawa et al. [18] showed that HIF1α activates Ror1 transcription in glioblastoma and affects cancer progression by regulating cell proliferation and migration. Other related factors studied include miR-210-3p by Agrawal et al. [19]; CXCR7, CXCR4, and IDH1 by Bianco et al. [20]; C-Met and SF/HGF by Eckerich et al. [21]; S100A4/NMIIA axis by Inukai et al. [22]; NO and VEGF by Kimura et al. [23]; BAG3 by Li et al. [24]; and tryptophan 2,3-dioxygenase (TDO2) by Mohapatra et al. [27].

Studies have shown that HIF1α and HIF2α orchestrate several cellular processes that are crucial for the pathogenesis of glioblastoma. For example, HIF1α is involved in promoting radioresistance by modulating AMPK-mediated ATM expression [16], promoting glioma invasion and stem cell formation via regulating MIR210HG and OCT1 [16], and activating Ror1 transcription to influence cancer progression [18]. In addition, HIF1α is involved in the regulation of miR-210-3p, CXCR7, CXCR4, IDH1, C-Met, SF/HGF, the S100A4/NMIIA axis, NO, VEGF, BAG3, and TDO2, and influences various aspects of the glioblastoma biology such as angiogenesis, invasion, metabolism, and therapy resistance. Similarly, HIF2α contributes to glioblastoma progression by regulating genes such as GPx1, vasorin, beclin-1, and galectin-3, thereby influencing the response to oxidative stress, angiogenesis, autophagy, and cell survival.

#### 3.2.2. Effect of Gene Modifications Related to HIFs

The collective results of various studies emphasize the multiple roles of HIFs and related factors in the pathogenesis of glioblastoma. For example, HIF1α was found to orchestrate regeneration resistance by upregulating AMPK-mediated ATM expression in severe hypoxia [16], while it promotes glioma invasion and stem cell formation by regulating MIR210HG and OCT1 [17]. In addition, HIF1α has been associated with the activation of Ror1 transcription to influence cancer progression [18], and it regulates miR-210-3p, CXCR7, CXCR4, IDH1, C-Met, SF/HGF, the S100A4/NMIIA axis, NO, VEGF, BAG3, and TDO2, and influences various aspects of the glioblastoma biology such as angiogenesis, invasion, metabolism and therapy resistance [19,20,21,22,23,24,25,26,27,28,30,31,32,33,34]. Similarly, HIF2α has been shown to regulate GPx1 to ensure resistance to oxidative stress and radiation [59], while contributing to glioblastoma progression via various mechanisms such as the DDX28-mediated regulation of eIF4E2-driven translation [49]. In addition, other factors such as miR-370-3p [61], NIX-mediated mitophagy [62], and p21 (CDKN1A)[63] have been identified as crucial players in glioblastoma pathogenesis, highlighting the complex interplay of genetic alterations in shaping the aggressive behavior of glioblastoma cells in the hypoxic tumor microenvironment.

### 3.3. Role of HIF-Related Targeted and Systematic Therapy of Glioblastoma

Table 3 shows a total of 26 laboratory studies addressing targeted and systemic therapies for glioblastoma in animal species with inoculated tumors or glioblastoma models. HIF1α was investigated in 25 of the 26 studies, while HIF2α was investigated in two studies.

A plethora of studies clarify the different roles of HIF1α in the progression of glioblastoma and response to treatment. Nardinocchi et al. [64] demonstrated the zinc-induced degradation of HIF1α, which inhibits VEGF-mediated signaling pathways and improves cancer therapies. Maugeri et al. [65] emphasized the role of HIF1α in angiogenesis via the upregulation of VEGF, while Ma et al. [66] linked the overexpression of HIF1α to glucose metabolism, suggesting its involvement in metabolic adaptations. D’Amico et al. [67] showed that ADNP modulates the HIF signaling pathway and reduces VEGF secretion and migration. In addition, D’Alessio et al. [68] pointed to antiangiogenic therapy targeting HIF1α and related factors to inhibit neoangiogenic events in glioblastoma.

Several related factors influence HIF1α-mediated signaling pathways in glioblastoma. These include VEGF, which is influenced by zinc, as shown by Nardinocchi et al. [64], and PA-CAP, as shown by Maugeri et al. [65], suggesting its role in regulating angiogenesis. Ma et al. [64] highlighted the association of HIF1α with glucose metabolism through the upregulation of GLUT-1, GLUT-3, and HK2. D’Amico et al. [67] revealed the modulation of the HIF signaling pathway by ADNP, reducing VEGF secretion and migration. Other factors such as M2 receptors, CXCR4, POL5551, LonP1, CT-L, PPARα, and SUMO are involved in regulating various aspects of glioblastoma progression and response to therapy, as noted by Cristofaro et al. [69], Gagner et al. [89], Douglas et al. [72], Hofstetter et al. [76], and Bernstock et al. [83]. In addition, Lin et al. [71] highlighted the far-reaching influence of HIF1α on tumor cell behavior, while Lin et al. [88] investigated the regulation of pH-regulatory proteins in glioblastoma by hypoxia-induced HIF1α. In the field of glioblastoma therapy, various targeted and systematic approaches have emerged to target the complex signaling pathways mediated by HIF1α. As noted by Nardinocchi et al. [64], zinc induces the proteasomal degradation of HIF1α and could thus prevent tumor progression by suppressing VEGF, MDR1, and Bcl2 signaling pathways. PACAP, identified by Maugeri et al. [65], is promising as it inhibits the release of VEGF and thus prevents the formation of new vessels in the hypoxic microenvironment of glioblastoma. Ma et al. [64] showed that acriflavine in combination with PDT effectively suppresses HIF1α expression and increases the efficacy of PDT against glioblastoma. D’Amico et al. [67] showed that ADNP can modulate the HIF signaling pathway to decrease VEGF secretion and migration, which is a targeted therapy approach. Gagner et al. [89] demonstrated the potential of the combination of B20-4.1.1 and POL5551 in reducing glioma invasion and tumor spread. As noted by Arienti et al. [73], HBO shows promise in inhibiting proliferation, downregulating HIF1α expression, and reprogramming glucose metabolism, offering the potential for the systemic therapy of glioblastoma.

### 3.4. Role of Combined Gene and Targeted or Systematic Therapy of Glioblastoma

A total of 23 studies used a combined gene-modifying design and targeted or systematic therapy in the context of HIF in laboratory glioblastoma models (Table 4). All studies investigated HIF1α, while five studies also investigated the role of HIF2α in addition to HIF1α. Gene modification techniques included transduction (N = 1; 4.3%), combined techniques (N = 4; 17.4%), transfection (N = 8; 34.8%), and knockdown (N = 10; 43.5%).

Several studies have elucidated the multiple roles of HIFs in the pathogenesis and therapy of glioblastoma. Huang et al. [90] showed that the PI3K/Akt/mTOR/HIF1α signaling pathway enhances glioblastoma cell migration and invasion under hypoxia, with mTOR pathway siRNA suppressing these effects. Chhipa et al. [91] showed that the activation of the AMPK/CREB1 axis supports glioblastoma cell bioenergetics by increasing HIF1α transcription. Pang et al. [92] highlighted the role of HIF1α-regulated lysosomal protease LGMN in TAMs and showed that its blockade prolongs survival in glioblastoma models. Hu et al. [113] identified HIF1α and AMPK as the regulators of hypoxia-induced LC3 changes, BNIP3 expression, and p62 degradation, which affect autophagy and responsiveness to bevacizumab. Barliya et al. [95] linked Hsp90 to angiogenesis, migration, and invasion, and highlighted its mediation of the HIF1α-driven signaling pathways. Hsieh et al. [103] demonstrated Nox4-mediated ROS production under cyclic hypoxia, which affects HIF1α activity and tumor growth. Kannappan et al. [97] showed that NF-kB/HIF1α/HIF2α promotes EMT and metastasis. Joseph et al. [98] elucidated the HIF1α-ZEB1 axis in mesenchymal transition and invasion. These findings emphasize the complex involvement of HIFs in glioblastoma progression and point to potential therapeutic targets.

Several drugs targeting HIF1α and related signaling pathways have been evaluated for their effects on glioblastoma. Huang et al. [90] showed that inhibitors such as 2-mercaptoethanol, LY294002, rapamycin, and p70S6K siRNA inhibited the PI3K/Akt/mTOR signaling pathway and suppressed migration, invasion, and HIF1α expression in glioblastoma cells. Chhipa et al. [89] showed that AMPK inhibitor (bafilomycin) decreased the viability of glioblastoma stem cells (GSCs), while Pang et al. [100] found that anti-PD1 antibody synergistically blocked the HIF1α-LGMN axis with anti-PD1 therapy in glioblastoma. Hu et al. [113] showed that BEV and chloroquine reversed hypoxia-induced growth by increasing BNIP3 expression and blocking autophagy, respectively, while Kannappan et al. [95] showed that disulfiram selectively targeted hypoxia-induced GSCs and digoxin inhibited HIF1α mRNA translation. Other drugs such as chlorpromazine, echinomycin, fenofibrate, and R50922/R59949 inhibit glioblastoma growth via several mechanisms, including the blockade of dopamine signaling, interference with the HIF1α-PDGFD/PDGFRα-AKT pathway, and the induction of apoptosis [99,100,101,102]. Tempol and YC-1 inhibit tumor growth by blocking ROS production and the induction of ABCB1, respectively [92,101]. In addition, digitoxin reduces HIF1α protein accumulation, while AMD3100 increases radiosensitivity by inhibiting SDF-1/CXCR4 interactions [105,111].

### 3.5. Role of HIFs in Clinal Studies of Glioblastoma

Nine studies have investigated the expression and clinical significance of HIFs in glioblastoma (Table 5). Chen et al. [114] found that HIF1α expression correlated with high caveolin-1 (CAV1) expression, larger glioblastoma size, and shorter survival time. Bache et al. [115] observed higher expression of HIF2α, carbonic anhydrase 9 (CA9), vascular endothelial growth factor (VEGF), and other markers in glioblastoma compared to tumor-free brain tissue, with mRNA levels correlating with shorter survival. Erpolat et al. [116] reported that high levels of cytoplasmic and nuclear HIF1α and CA9 were associated with shorter survival, especially in patients with high hypoxia scores. Clara et al. [115] found that HIF1α expression correlated with increased vascular density, VEGF, and platelet-derived growth factor-C (PDGF-C) and survival. Other studies, such as those by Kaynar et al. [117] and Nobuyuki et al. [118], also emphasized the role of HIF1α in angiogenesis and radioresistance in glioblastoma. In addition, Ji et al. [119] showed that high HIF1α expression correlates with poorer outcomes and shorter survival, suggesting its potential as a prognostic marker. Sfifou et al. [120] found that negative HIF1α expression in conjunction with the positive expression of isocitrate dehydrogenase 1 (IDH1) was associated with a better prognosis. Potharaju et al. [121] observed the strong nuclear staining of HIF1α in a significant proportion of samples, which independently correlated with poor prognosis, especially in combination with the high expression of telomerase reverse transcriptase (TERT).

### 3.6. Common HIF-Related Pathways in Glioblastoma

**Figure 5:**
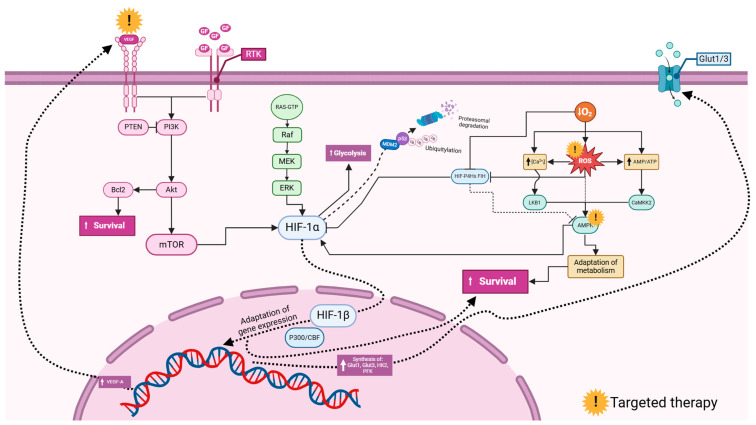
Commonly investigated signaling pathways involving hypoxia-inducible factors (HIFs) in glioblastoma. Vascular endothelial growth factor (VEGF) and growth factors (GFs) activate Receptor Tyrosine Kinases (RTKs), triggering downstream signaling. The PI3K (Phosphoinositide 3-Kinase) pathway, inhibited by PTEN (Phosphatase and Tensin Homolog), activates Akt, leading to enhanced cell survival via the Bcl2 inhibition of apoptosis and mTOR (mechanistic target of rapamycin) promotion of growth. The RAS/MAPK (Mitogen-Activated Protein Kinase) pathway, through Raf, MEK, and ERK, also supports cell proliferation and survival. HIF-1α (hypoxia-inducible factor 1-alpha) under hypoxia increases glycolysis for energy production and gene expression for adaptation, regulated by HIF-1β (hypoxia-inducible factor 1-beta). HIF-1α stability is controlled by ubiquitination via MDM2 (Mouse Double Minute 2) and proteasomal degradation influenced by p53. Reactive oxygen species (ROS) and Calcium ion (Ca^2+^) signaling activate survival pathways, involving LKB1 (Liver Kinase B1), AMPK (AMP-activated Protein Kinase), and CaMK2 (Calcium/Calmodulin-Dependent Protein Kinase II). Glucose transporters Glut1/3 facilitate glucose uptake. This network highlights potential therapeutic targets, such as mTOR, PI3K, and HIF-1α, to disrupt glioblastoma cell survival and adaptation mechanisms. The figure was created using the BioRender online commercial platform.

Glioblastoma involves a complex interplay of molecular signaling pathways, among which the PI3K/Akt/mTOR pathway stands out. This signaling pathway exerts a profound influence on the progression of glioblastoma and modulates important cellular processes such as migration, invasion, and the expression of HIF1α. The importance of this pathway is further emphasized by the fact that it can be modulated by PTEN-PI3K interactions, offering potential therapeutic opportunities (Figure 5). The intricate relationship between HIF1α and metabolic pathways adds another layer of complexity. HIF1α not only affects glucose metabolism by upregulating the glucose transporters GLUT-1 and GLUT-3 but also enhances glycolysis through the overexpression of hexokinase 2 (HK2). This metabolic switch contributes to the robustness of glioblastoma cells and allows them to thrive in the hypoxic tumor microenvironment. In addition, the therapeutic landscape in glioblastoma is evolving with the emergence of new strategies targeting HIF1α-related axes. The synergistic blockade of the HIF1α-LGMN axis, aided by AMPK inhibition and anti-PD1 antibody therapy [92], represents a promising approach to interrupting glioblastoma progression. Furthermore, interventions targeting VEGF [64,67,68,84,86], such as digoxin, offer potential opportunities to inhibit angiogenesis and overcome multidrug resistance (MDR) mediated by pathways involving Bcl2 [64]. Understanding and interfering with these pathways are key to developing more effective treatments for glioblastoma, a disease with poor prognosis and limited therapeutic options.

## 4. Discussion

### 4.1. Research Trends

The high morbidity and mortality rate of glioblastoma has led to conventional treatments such as surgery, radiotherapy, and chemotherapy being re-evaluated due to their limited effectiveness. Researchers around the world, particularly in the United States and China, are exploring new treatments and incorporating molecular genetic features into diagnostics to better understand the pathogenesis of glioblastoma [2,124]. Despite an increase in in vitro and in vivo studies focusing on hypoxia-regulated genes, clinical trials remain limited, accounting for only 9.6% of the total. Advances in diagnostic methods, particularly next-generation sequencing, have led to significant growth in research [125,126]. However, the translation of promising laboratory results into clinical practice is challenging due to small sample sizes and geographic variation, making it difficult to develop standardized global diagnostic and treatment algorithms [66,116,121,123].

### 4.2. The Impact of HIF-Related Gene Modification on Glioblastoma Therapeutics

The importance of the knockdowns and knockouts of hypoxia-inducible factors lies in their ability to reveal the precise roles and functions of these factors in cellular processes and disease progression [127]. By elucidating the effects of manipulating hypoxia-inducible factors on glioblastoma progression, these techniques provide insights into potential therapeutic targets. Key findings include the functional importance of the interaction of N-cadherin and β-catenin on the radioresistance of glioblastoma stem cells. Elevated glucose-6-phosphatase (G6PC) levels contribute to resistance to glycolytic inhibition in glioblastoma cells [128]. AMPKα1 knockout affects glycolysis and tumorigenesis in a lymphoma mouse model. The overexpression of HHIF2α in AMPK knockdown GSCs possibly compensates for the loss of HIF1α. AMPKα knockdown decreases the expression of Sp1 and ATM under severe hypoxia and reduces radioresistance [91]. Moreover, the overexpression of MIR210HG enhances IGFBP2 and FGFR1 promoter activities under normoxia, which is inhibited by the suppression of OCT1, and decreases under hypoxia with MIR210HG or OCT1 knockdown. These findings emphasize the multifaceted role of hypoxia-inducible factors in glioblastoma, which includes radioresistance, migration, the regulation of gene expression, and metabolic processes [17].

Laboratory-based studies, such as those listed in Table 3, involve experimental manipulations and investigations performed on cells or animal models. This controlled environment allows researchers to isolate specific mechanisms, control variables, and collect preliminary data on the effects of hypoxia-inducible factors on glioblastoma. However, human clinical trials are challenging due to ethical considerations, difficulties in obtaining tumor samples, the heterogeneity of the patient population, and the complexity of studying hypoxia-inducible factors in the clinical setting [129]. Although laboratory-based studies provide valuable insights, they cannot fully reflect the complexity of human glioblastoma. Therefore, further research with human clinical trials is essential to validate the laboratory results and determine the clinical significance of hypoxia-inducible factors in glioblastoma.

### 4.3. Exploring HIF-Related Targeted and Systemic Therapies for Glioblastoma in Experimental Settings

Given the central role of HIF-1 in the pathophysiology of glioblastoma, the identification of a specific HIF-1 inhibitor holds promise for overcoming resistance to cytotoxic therapy and improving overall survival. Zinc is a potential candidate, as shown by Nardinocchi et al. [64], who observed its ability to induce the proteasomal degradation of HIF1α. While zinc showed similar effects in prostate cancer under hypoxic conditions, its efficacy was not present in the human RCC4 VHL-null cell line. Meanwhile, Maugeri et al. [65] found that PACAP inhibited the release of VEGF. D’Amico et al. [67] showed that this inhibition occurs through the activation of ADNP, a protein that is central to normal brain development and plays a dual role as an oncogene or tumor suppressor, depending on the tumor type. Although the involvement of PACAP in neurodegenerative diseases is well established, further investigation of the PACAP-ADNP axis in glioblastoma is warranted.

Another strategy for inhibiting VEGF is the use of BEV, an anti-VEGF monoclonal antibody that is frequently used in the treatment of glioblastomas. Preclinical and clinical studies have consistently shown that BEV is able to prolong progression-free and overall survival. However, a major challenge is to identify the patients who would benefit from this therapy, as many of them quickly develop resistance. This challenge is exacerbated by the lack of reliable biomarkers, as D’Alessio et al. [68] point out.

Despite BEV treatment, a significant proportion of glioblastoma cases (40–60%) continue to progress, as shown in the clinical studies by Hu et al. [113]. Ongoing randomized clinical trials are investigating the potential of combining chloroquine with the standard treatment of glioblastoma, but a significant benefit has not yet been demonstrated.

In a 2017 study, Gagner et al. [70] used glioma models with mice and administered the anti-VEGF antibody B20-4.1.1 and showed reduced tumor invasiveness in combination with POL5551, a CXCR4 antagonist previously shown to improve survival in immunodeficient mice when combined with other therapeutic modalities. Clinical trials with various CXCR4 antagonists are ongoing. For example, the study (NCT01339039) combines BEV with AMD3100 in patients with recurrent high-grade glioma, while another study (NCT01837095) is investigating POL6326 in combination with the chemotherapeutic agent eribulin in patients with metastatic breast cancer. Kioi et al. [111] investigated the SDF-1/CXCR4 inhibitor AMD3100 and reported its superior efficacy over VEGF blockade in reducing tumor tissue perfusion after radiotherapy.

Photodynamic therapy (PDT) has impressive complete remission rates of up to 90% for skin, head, and neck tumors as well as for early-stage lung and bladder cancer. However, the efficacy of PDT in the treatment of glioblastoma has been limited in the past. However, recent advances, such as the use of acriflavine (ACF) to inhibit HIF1α, as shown by Ma et al. [66], are promising. ACF, which is known for its safety profile, has extended median survival in patients with glioblastoma to 21 months after diagnosis. Since PDT usually upregulates HIF1α expression in most tumors, the integration of HIF inhibitors is crucial. Li et al. [108] have shown that PDT enhances the effect of TMZ by suppressing glycolytic metabolism. The role of immune cells and glycolysis-related enzymes should be further explored.

Hyperbaric oxygen therapy (HBO), which is used in the treatment of ischemic diseases, is also used in carcinoma therapy alongside radiotherapy [130]. Arienti et al. [73] demonstrated that HBO can inhibit the proliferation of glioma cells by increasing reactive oxygen species, which leads to DNA damage. However, preclinical studies often provide contradictory results. For example, Chen et al. [131] report the antitumor effects of HBOT, while there is evidence of tumor-promoting effects [132]. Although clinical studies support the use of HBOT as an adjunct to radiotherapy, a scientific rationale for this phenomenon remains elusive.

Cardiac glycosides that are effective in the treatment of malignancies have been identified as HIF1α inhibitors. The studies by Bar et al. [106], Joseph et al. [98], and Papale et al. [106] highlight the efficacy of digoxin, while Lee et al. [40] focused on digitoxin due to its liposolubility, suggesting the possible permeability of the blood–brain barrier.

Fenofibrate, known for the treatment of hyperlipidemia, has an anticancer effect that has been demonstrated in melanoma, medulloblastoma, and GBM. Trejo-Solis et al. [133] demonstrated its inhibition of glycolysis in GBM, while Lin et al. [71] elucidated the HIF1α inhibition of fenofibrate via multiple metabolic pathways. 2-Methoxyestradiol (2ME2) inhibits HIF1α, inhibits tumor growth, and is being tested in phase I and II in various cancers, including GBM, with promising efficacy and low toxicity. However, the development of resistance to 2ME2 remains enigmatic. Muh et al. [85] suggest PTEN analysis to predict patient response. Combination therapy with a PI3K inhibitor, such as LY294002, is suggested for improved efficacy.

In their effort to target glioma cell proliferation and improve the efficacy of TMZ, Douglas et al. [72] directed their research towards identifying a compound with the dual inhibition of LonP1 and CT-L. BT317 emerged as a promising candidate due to its ability to penetrate the blood–brain barrier, its low toxicity in animals, and its improved survival rates. However, in vivo tests with ritonavir led to the rapid development of resistance. In contrast, marizomib showed significant CNS toxicity in phase II studies and no improvement in survival was demonstrated in phase III trials. Hofstetter et al. [76] found that the inhibition of PP2A with LB1.2 enhanced the effect of TMZ on GBM and neuroblastoma in mouse studies, with no side effects observed during short-term monitoring.

Borneol, a terpene from traditional Chinese medicine, sensitizes cells to TMZ by promoting HIF1α degradation, as demonstrated by Lin et al. [88]. Previous studies have also shown that borneol enhances the efficacy of doxorubicin [134], curcumin [135], cisplatin [136], and radiotherapy [137]. Liu et al. [79] demonstrated in preclinical studies the usefulness of mannose as an adjunct to TMZ and to enhance radiotherapy, and achieved long-term survival in mice.

By combining methoxyamine and resveratrol with iododeoxyuridine, Khoei et al. [78] increased the sensitivity of GBM to radiotherapy. Ahmed et al. [104] noted that the sensitivity of GBM to cisplatin under hypoxic conditions may be independent of HIF and may be induced by the activation of CD133. Barliya et al. [95] investigated the effects of hypericin on the degradation of hsp90 and HIF1α in GBM and renal cell carcinoma cells, with modest results from phase I and phase II trials.

Hsieh et al. [103] reported the inhibition of HIF-1 activation and tumor growth by tempol, while Chou et al. [94] investigated the ability of YC-1 to enhance the efficacy of chemotherapy BCNU. Although not specific to HIF1, Chen et al. [114] demonstrated the synergistic effect of YC-1 with Bay 11-7082 by inhibiting Bcl-xL induction under hypoxia-induced TMZ resistance.

TAT-Lp15, a livin peptide inhibitor, sensitized GBM cells to radiotherapy and TMZ without affecting healthy tissues, as shown by Hsieh et al. [103]. In particular, the ability of TAT-Lp15 to cross the blood–brain barrier underscores its therapeutic potential and warrants further clinical validation.

Disulfiram, known for its ability to improve the efficacy of standard chemotherapies in various carcinomas while exhibiting low toxicity to healthy cells, is hampered by its short half-life in the bloodstream. To address this problem, Kannappan et al. [97] investigated DS-PLGA, an intravenously administered formulation that prolongs the residence time of disulfiram in the bloodstream and facilitates its penetration into GBM tissues without adverse effects on vital organs.

Sulfinosine (SF), known for its multiple anticancer effects via different metabolic pathways, has the potential to prevent cancer cells from developing resistance [138]. Dačević et al. [80] investigated the effect of SF in small-cell lung cancer and GBM and emphasized its ability to penetrate the CNS and its compatibility with other chemotherapeutic agents. Topotecan, which is approved for cervical, ovarian, and small-cell lung cancers, acts as both a DNA topoisomerase I inhibitor and a HIF1α inhibitor [139]. However, its efficacy in GBM remains limited, as noted by Bernstock et al. [83]. Nelfinavir and amprenavir, which have been shown to be effective in HIV therapy, inhibit both HIF1α and VEGF and could sensitize tumor cells to radiotherapy with minimal toxicity, as shown by Mait et al. [86].

Dominguez et al. [102] have identified DGKα as a promising therapeutic target for GBM and other carcinomas, with selective toxicity observed in malignant GBM cells when treated with the DGKα inhibitors R59022 and R59949. SGC707, a PRMT3 inhibitor, showed anticancer activity in GBM by inhibiting HIF1α and glycolysis while sparing normal brain cells, as found by Liao et al. [110].

Arecaidine propargyl ester (Ape) activates M2 muscarinic receptors, leading to cell cycle arrest in GBM stem cells, as reported by Cristofaro et al. [67]. WIN 55,212-2, a cannabinoid receptor agonist, induces GBM cell death, suggesting cannabinoids as potential anticancer agents according to Sugimoto et al. [87]. Paris saponin H, which is used in the treatment of lung cancer and malignant lymphoma, induces the apoptosis of gliomas, as shown by Bi et al. [77]. Although the insulin signaling pathway plays a crucial role in the progression of GBM, drugs targeting IGF1 await the successful completion of phase III trials as the molecular mechanisms involved are not yet fully understood, as noted by Lin et al. [71]. Echinomycin, a notable HIF1α inhibitor, induces apoptosis and inhibits GBM growth by targeting the HIF1α-PDGFD-PDGFRα axis, as found by Peng et al. [100].

### 4.4. Insights into HIF-Associated Discoveries from Clinical Investigations in GBM

Clinical studies consistently report the elevated expression of HIF1α in glioblastoma (GBM) tissues, suggesting its pivotal role in tumor progression. Chen et al. (2019) [114] observed significant HIF1α expression in both the nucleus and cytoplasm of GBM cells, correlating with tumor vasculature, indicating its involvement in angiogenesis. Similarly, the findings by Carlos Alfonsoe et al. [122] and Xiangjun et al. [119] linked HIF1α expression in GBM with increased vascular proliferation and poorer patient prognosis. Moreover, the research by Bache et al. [115] and El-Benhawy [123] described a diverse range of hypoxia-related factors, including HIF2α and OPN, contributing to the intricate tumor microenvironment, highlighting the multifaceted role of HIFs in tumor growth and survival under hypoxia.

Notably, the studies by Erpolat et al. [116] and Nobuyuki et al. [118] established a correlation between elevated HIF1α levels and reduced patient survival, indicating its potential as a prognostic marker. Conversely, the observations by Sfifou et al. [120] indicated longer survival in patients with negative HIF1α expression, reinforcing its prognostic value. High HIF expression levels correlate with aggressive GBM behavior, including rapid growth, enhanced invasiveness, and resistance to standard treatments, as demonstrated by Kaynar et al. [117] and Potharaju et al. [121], contributing to poorer patient outcomes.

These clinical findings underscore the importance of investigating hypoxia-induced tumor progression mechanisms in GBM. Developing targeted therapies to inhibit HIF activity, possibly in combination with existing treatments, holds promise for improving patient prognosis. Additionally, identifying novel biomarkers based on hypoxia-related factors could enhance early detection and treatment monitoring in GBM, ultimately improving patient outcomes. Future research efforts should focus on unraveling the complexities of the hypoxic tumor microenvironment to devise more effective interventions for managing GBM.

### 4.5. Advantages, Disadvantages, and Future Directions

Therapies targeting HIFs offer a promising avenue for combating GBM, a malignancy notorious for its resistance to conventional treatments. By specifically inhibiting HIF activity, these therapies hold potential for improving patient outcomes, particularly in cases where GBM displays elevated HIF expression levels [140]. Combining HIF-related therapies with established treatments like surgery, radiation, and chemotherapy may enhance their effectiveness, offering a more comprehensive approach to GBM management [141,142]. Research into HIFs in GBM provides crucial insights into tumor progression mechanisms, offering hope for the development of more potent therapeutic strategies. Moreover, exploring HIFs could lead to the identification of novel biomarkers for early diagnosis, prognosis assessment, and treatment response monitoring in patients with GBM [143].

However, challenges abound in the clinical application of HIF-related therapies. The lack of standardization in research methodologies impedes quantitative meta-analysis, while genetic mutations in GBM and therapy effects outside target sites present additional hurdles [13,144,145]. The complex and dynamic nature of the hypoxic tumor microenvironment may limit the efficacy of single-target HIF therapies, potentially leading to therapy resistance. Developing combination therapies or innovative treatment strategies may be necessary to address this issue. Despite encouraging preclinical results, limited clinical data exist on the efficacy of HIF-related therapies in patients with GBM, necessitating further extensive clinical trials for validation [137,146]. Safety concerns, including potential side effects and toxicity, especially when combined with other treatments, require thorough evaluation [147,148,149].

Moreover, the challenge lies in targeting HIFs without disrupting normal cellular responses to hypoxia, underscoring the need for precision in therapy development [150]. The absence of reliable biomarkers to identify patients who would benefit most from HIF-related therapies complicates treatment decisions and personalized care plans. Exploring combination therapies targeting multiple GBM progression pathways, conducting advanced clinical trials with diverse populations, and investigating the mechanisms of therapy resistance are crucial steps forward [151]. Additionally, advancing research to identify and validate biomarkers for early detection and treatment response monitoring is essential for the effective clinical implementation of HIF-related therapies in GBM management.

## 5. Conclusions

In conclusion, the evolving landscape of GBM research reflects a concerted effort to address the pressing challenges of poor patient outcomes associated with conventional treatments. While molecular genetic features have improved diagnostic capabilities, preclinical studies have highlighted the importance of HIFs as a therapeutic target, although clinical translation is limited. Overcoming challenges such as therapy resistance, safety concerns, and the absence of reliable biomarkers is crucial for the successful integration of HIF-related therapies into the treatment of GBM. By combining targeted approaches with conventional treatments, conducting large clinical trials, and testing combination therapies, researchers aim to optimize patient outcomes and pave the way for personalized treatment strategies in GBM. Ultimately, these multidisciplinary efforts promise to improve our understanding and treatment of GBM and provide hope for better patient care in the future.

## Figures and Tables

**Table 1:** PICOS strategy.

Acronym	Search Strategy
P (population or problem)	Glioblastoma
I (intervention)	Hypoxia-inducible factors
C (comparison)	None
O (outcome)	None
S (study design)	Original investigations

**Table 2:** Laboratory studies with gene modification of HIFs in glioblastoma models and inoculated animals.

Reference	Country (Year)	Study Design	Species	Cell Line(s)	Targeted HIF	Related Factor	Role of HIF and Related Factors	Gene Modification	Effect of Gene Modification
Hashimoto et al. [16]	Japan (2022)	Lab (IV)	CL	T98G and A172	HIF1α	AMPK and ATM	AMPK boosts ATM expression via Sp1 transcription factor, eliciting radioresistance in severe hypoxia.	KD	AMPKα KD under severe hypoxia decreases Sp1 and ATM expression, whereas Sp1 KD suppresses ATM, Src, EGFR, and Akt expression, ultimately diminishing radioresistance.
Ho et al. [17]	Taiwan (2021)	Lab (C)	Mice and CL	U-87, U-118, and PDM-123	HIF1α	MIR210HG, OCT1, IGFBP2, and FGFR1	MIR210HG participates in hypoxia-mediated glioma invasion, cancer stemness, and TMZ resistance. It also promotes the transcription activity of OCT1, regulating the expressions of the oncogenes IGFBP2 and FGFR1.	KD	The overexpression of MIR210HG in normoxia boosts the activities of IGFBP2 and FGFR1 promoters, an effect that is inhibited by the suppression of OCT1. In hypoxia, the promoter activities of IGFBP2 and FGFR1 are reduced when MIR210HG or OCT1 is knocked down.
Ishikawa et al. [18]	Japan (2022)	Lab (IV)	CL	T98G, A172, and U87	HIF1α	Ror1 (Wnt5a-Ror1 axis)	HIF1α activates Ror1 transcription by binding to its promoter regions in glioblastoma, influencing cancer progression via cell proliferation and migration regulation.	KD	KD of HIF1α inhibited the expression of Ror1, in particular under hypoxic conditions.
Agrawal et al. [19]	India (2014)	Lab (IV)	CL	U251, U87, and A172	HIF1α	miR-210-3p	miR-210-3p promotes the survival, aggressiveness, and therapy resistance of glioblastoma cells. The regulation of miR-210-3p is HIF1α dependent and, on the other hand, miR-210-3p promotes HIF transcriptional activity.	OE	Increase in the expression of the HIF target genes VEGF and CA9 in response to miR-210-3p overexpression and their downregulation in response to miR-210-3p inhibition.
Bianco et al. [20]	Brazil (2015)	Lab (IV)	CL	U87	HIF1α	CXCR7, CXCR4, and IDH1	CXCR7 expression in astrocytoma varies with malignancy; HIF1α boosts CXCR7 and CXCR4, whereas IDH1mut lowers them, suggesting CXCR7 involvement in astrocytoma tumorigenesis.	OE	HIF1α overexpression was linked to higher CXCR7 and CXCR4 expressions, while IDH1 mutation was associated with lower levels; CXCR7 overexpressed in astrocytoma and correlated with CXCR4/IDH1 in AGII and with CXCR4/IDH1/HIF1α in glioblastoma, with no survival correlation.
Eckerich et al. [21]	Germany (2007)	Lab (IV)	CL	U87 and U251	HIF1α	C-Met and SF/HGF	SF/HGF, a multifunctional growth factor, binds to c-Met, a tyrosine kinase receptor encoded by a proto-oncogene; hypoxia activates the c-met promoter containing HIF-1 binding sites.	KO	Half of all human glioblastomas respond to hypoxia with an induction of c-Met, which can enhance the stimulating effect of SF/HGF on tumor cell migration.
Inukai et al. [22]	Japan (2022)	Lab (IV)	Mice and CL	KS-1	HIF1α	S100A4/NMIIA axis	Following severe hypoxia, S100A4 is upregulated and interacts with NMIIA; this inhibits NMIIA activity and thus derepresses tumor cell migration.	KD	The KD of S100A4 in the glioblastoma cell line KS-1 decreased migration capability, concomitant with decreased Slug expression.
Kimura et al. [23]	Italy (2000)	Lab (IV)	CL	A172 and Hep3B	HIF1α	NO and VEGF	The direct involvement of NO in the control of angiogenesis through its regulation of VEGF expression, where HIF1α activity appears to be essential.	DEL	NO-responsive cis-elements are HIF1α binding sites, and an adjacent ancillary sequence is located immediately downstream within the hypoxia-response element (HRE).
Li et al. [24]	China (2018)	Lab (IV)	CL	U87 and U251	HIF1α	BAG3	Downregulated BAG3 inhibited HIF1α protein through promoting the degradation of HIF1α by HSP70 by the BAG3/HSP70/HIF1α proteasome pathway.	TF	When HIF1α was upregulated, induced by HIF1α plasmid TF based on the downregulation of BAG3, the proliferation inhibition and apoptosis promotion was partially reversed.
Mendez et al. [25]	USA (2010)	Lab (C)	Mice and CL	LN308, U87MG, HEK 293T, and GL261	HIF1α	n/a	HIF1α plays a role in the survival and self-renewal potential of CSCs.	KD	The KD of HIF1α in human and murine glioma cells impairs their migration in vitro and their invasion in vivo.
Miska et al. [26]	USA (2019)	Lab (C)	Mice and CL	Biopsy	HIF1α	Foxp3+ T Cells	HIF1α acts as a metabolic switch for Tregs between glycolytic-driven migration and oxidative phosphorylation-driven immunosuppression.	KO	The conditional KO of HIF1α in Foxp3+ T Cells inhibits the migration of Tregs to brain tumors in vivo.
Mohapatra et al. [27]	Germany (2019)	Lab (IV)	CL	A172 and U-87 MG i LN-18	HIF1α	Tryptophan-2,3-Dioxygenase (TDO2)	TDO2 in glioblastoma promotes tumor cell motility and suppresses antitumor immune responses by producing Trp metabolites that activate the aryl hydrocarbon receptor (AHR).	KD	The KD of HIF1α restored the expression of TDO2 upon cobalt chloride treatment, confirming that HIF1α controls TDO2 expression.
Mongiardi et al. [28]	Italy (2016)	Lab (IV)	CL	U87	HIF1α	c-MYC	HIF-1 and a deregulated c-MYC in cancer cells cooperatively induce the transcription of genes involved in hypoxic adaptation such as genes regulating metabolic reprogramming and angiogenesis.	TD	MYC inhibition alters the transcriptional response to hypoxia in glioblastoma cells.
Nie et al. [29]	China (2012)	Lab (IV)	CL	U87, U251, U118, LN229, and SHG44	HIF1α	Casein kinase 1α 1	CK1a is overexpressed in glioblastoma cells, with its levels increasing proportionally with the WHO grade.	TF	Overexpressed CK1a positively regulates autophagy activity through the HIF1α pathway. The inhibition of CK1a might be a potential therapeutic approach for glioblastoma therapy.
Noch et al. [30]	United States (2011)	Lab (C)	Mice and CL	U87 and T98-G	HIF1α	Astrocyte-elevated gene-1 (AEG-1)	The hypoxic induction of AEG-1 relies on HIF1α stabilization, with PI3K inhibition disrupting AEG-1 induction by destabilizing HIF1α.	TF	AEG-1 is slightly upregulated following 24 h TF with HIF1α.
Pistollato et al. [31]	Italy (2009)	Lab (IV)	CL	Biopsy	HIF1α	Akt/mTOR and BMP2	Exogenous BMP2, similar to high oxygen exposure, induces the time-dependent activation of the Akt/mTOR pathway in glioblastoma-derived cells.	KD	By silencing HIF1α in glioblastoma cells, a strong differentiation and eventually cell death occurred after 1 week.
Qiang et al. [32]	China (2011)	Lab (IV)	CL	U251, SHG44, A172, and C6	HIF1α	PI3K/Akt and ERK1/2	PI3K/Akt and ERK1/2 pathways contribute to HIF1α translation in GSCs.	KD	PI3K/Akt and ERK1/2 inhibition partly reduces hypoxia-induced Notch pathway activation and GSC maintenance.
Said et al. [33]	Germany (2012)	Lab (IV)	CL	U373, U251, and U87	HIF1α	ndrg1 N-Myc	Short dsRNA oligonucleotides and iodoacetate inhibit N-Myc downregulated gene 1 protein and mRNA expression in U373 glioblastoma cells by interfering with cellular glycolysis.	KD	Treatment with siRNA and iodoacetate (IAA) in human glioblastoma cell lines led to a nearly complete suppression of NDRG1 expression, highlighting IAA’s role as a glycolysis inhibitor.
Sesen et al. [34]	France (2014)	Lab (IV)	CL	LN18, SF767, U87, and U251	HIF1α and HIF2α	Int6/eIF3e	siInt6 significantly inhibits Int6 mRNA and protein in all glioblastoma cell lines compared to control siRNA.	TF	TF silenced the Int6 gene and protein expression effectively.
Rong et al. [35]	United States (2006)	Lab (IV)	CL	U87 and U251	HIF1α	Egr-1, Sp1, NF-κB, and activator protein-1 (AP-1)	Forced Egr-1 overexpression, but not Sp1, via cDNA TF, increases tissue factor in glioma cells under normoxia (21% O2), while Egr-1 siRNA notably decreases hypoxia-induced tissue factor expression.	TF	The TF of glioma cells with an Sp1 expression plasmid (pSp1, 2.0 μg) for 24 h under normoxia led to a large increase in both nonphosphorylated (bottom band) and phosphorylated (top band) Sp1 protein expression without a concomitant tissue factor expression.
Fan et al. [36]	China (2021)	Lab (C)	CL	PN 12,16 and 19, MES23, 27 and 29	HIF1α	IDH1, TGF-β1, E2F4, and Smad3	IDH1 mutation activates HIF1α and reduces TGF-β1 expression in proneural GSCs; Smad3 interacts with E2F4 to inhibit the expression of mesenchymal markers.	KD	IDH1 KD elevates HIF1α and decreases TGF-β1 in proneural glioblastoma cells.
Voss et al. [37]	USA (2020)	Lab (C)	Mice and CL	HSR-GLIOBLASTOMA1, HSR-040821, HSR-040622, T387, T3691, and T3832	HIF1α and HIF2α	MBNL1	MBNL1 expression is highest in glioblastoma defined as MES, inhibited in the hypoxic elements of the tumor and within the MES subgroup, and correlates with better overall patient survival.	KD	Hypoxia suppresses MBNL1 activity in certain tumor-derived neurosphere lines, leading to the increased expression of various gene isoforms that are linked to an ESC-like state.
Wang et al. [38]	China (2021)	Lab (C)	Mice and CL	MES02-GSC, MES06-GSC, and MES13-GSC	HIF-1	PLOD1	HIF1 can directly induce the expression of PLOD1 under hypoxia.	KO	PLOD1 KO inhibits MES GSC-enriched tumor sphere growth and invasion in vitro, and differentiation in vivo.
Bae et al. [39]	South Korea (2021)	Lab (C)	Mice and CL	U87, T98G, H4, U251, immortalized primary human fetal astrocytes, and HMEC-1	HIF1α	Arrb2 (β-arrestin 2)	Arrb2 interacts with HIF1α and stimulates the ubiquitin-mediated 26S proteasomal degradation of HIF1α by recruiting PHD2 and pVHL.	TF	The overexpression of Arrb2 in glioblastoma cells reduces HIF1α levels, resulting in antitumorigenic effects including suppressed tumor growth and angiogenesis.
Feng et al. [40]	China (2019)	Lab (C)	Mice and CL	U251, U87, and HEK293	HIF1α	ANKDD1A	ANKDD1A inhibits HIF1α activity, decreases its half-life by upregulating FIH1, reduces glucose uptake and lactate production, inhibits glioblastoma autophagy, and induces apoptosis in glioblastoma cells under hypoxia.	TF	Transfected cells had lower glucose uptake and lower LDH. ANKDD1A disturbs the tolerance of glioblastoma cells to hypoxia.
Nishikawa et al. [41]	Japan (2021)	Lab (C)	Mice and CL	GSL-1 and GSL-2	HIF1α and HIF2α	CD44 and OPN	Hypoxia (1% O2) upregulates CD44 expression via the activation of HIF1α. Moderate hypoxia (5% O2) upregulates osteopontin expression via the activation of HIF2α.	KD	The upregulated osteopontin inhibits CD44-promoted GSC migration, invasion, and proliferation.
Choksi et al. [42]	USA (2012)	Lab (C)	Mice and CL	TRAF2−/−, wt MEF, A172, IMR-32 and CCF-STTG1	HIF1α	ATIA	HIF-1 target, ATIA protects cells against TNFα- and hypoxia-induced apoptosis through regulating the function of the mitochondrial antioxidant, thioredoxin-2, and ROS generation.	KD, KO	ATIA KD in glioblastoma cells renders them sensitive to hypoxia-induced apoptosis.
Lee et al. [43]	Korea (2022)	Lab (C)	Mice and CL	U251-MG, LN215-MG, CRT-MG, U373-MG, HT-1080 and Panc-1	HIF1α	Notch1	HIF1α, induced even in non-hypoxic conditions by cell-to-cell contact, is a critical cue responsible for the malignant characteristics of glioblastoma cells through Notch1 signaling.	TF	Silencing Notch1 signaling with siRNA TF resensitized resistant glioblastoma cells to TMZ and reduced their viability under high-density culture conditions.
Katakowski et al. [44]	USA (2016)	Lab (C)	Mice and CL	U87	HIF1α	miR-9	miR-9 increases glioma cell migration and decreases proliferation at low densities, but has the opposite effect at high densities.	TF	miR-9 has a biphasic density-dependent effect on glioma cell proliferation.
Ji et al. [45]	China (2014)	Lab (C)	Mice and CL	U251 and U87	HIF1α	Nrf2	Nrf2 has a role in glioblastoma angiogenesis; human glioblastoma tissues expressing higher Nrf2 levels showed relatively higher microvessel density.	KD	The KD of Nrf2 inhibits glioblastoma angiogenesis by preventing the hypoxia-induced activation of HIF1α.
Gauthier et al. [46]	France (2020)	Lab (C)	Mice and CL	TG1N i TG16 GSC	HIF1α	JMY	Post-irradiation, HIF1α induces JMY transcription, promoting GSC migration via its actin nucleation-promoting activity.	KD	The radiation-induced migration of GSCs is associated with the HIF1α-dependent accumulation of JMY in the cytoplasm.
Hu et al. [47]	China (2019)	Lab (IV)	CL	U87, U251, T98, LN229, and U118	HIF1α	miR-576-3p	miR-576-3p’s inhibition of the migration and proangiogenic capacity of hypoxia-induced glioma cells is mediated by HIF1α.	KD, TF	HIF1α KD and miR-576-3p overexpression comparably inhibit migration and angiogenesis in hypoxia-induced glioma cells, with reduced HIF1α expression in miR-576-3p-transfected cells.
Ghosh et al. [48]	India (2013)	Lab (IV)	CL	T98G and U87	HIF1α	TNF-α, β-catenin, and MHC 1	A TNF-α-induced increase in MHC-I expression and transcriptional activation was concurrent with increased HIF1α, ΝF-κΒ, and β-catenin activities.	KD	The KD of HIF1α and β-catenin abolished TNF-α-induced MHC-I activation, while NF-κB had no effect.
Evagelou et al. [49]	Canada (2020)	Lab (IV)	CL	U87	HIF2α	DDX28	HIF2α is responsible for regulating eIF4E2-directed translation in hypoxia, whereas DDX28 functions as a negative regulator, hindering HIF2α‘s ability to activate this translation pathway.	KD	eIF4E2 binds to the m7GTP cap structure, enhancing the translation of its target mRNAs, while the repression of HIF2α and eIF4E2 curtails the translation activation of oncogenic mRNAs.
Ikemori et al. [50]	Brasil (2014)	Lab (C)	Mice and CL	NG97ht, T98G, and U87G	HIF1α	Galektin-3 (gal-3)	Gal-3 expression shields glioma cells from hypoxia-induced death and facilitates tumor growth in poorly perfused microenvironments.	KD	The KD of Gal-3 enhances cell death in cells deprived of both oxygen and serum.
Man et al. [51]	USA (2018)	Lab (C)	Mice and CL	GSCs and non-GSCs	HIF1α	Vasorin	Vasorin prevents TNF-mediated apoptosis, inhibits TGF-beta signaling, and regulates Notch signaling in GSCs within the hypoxic niche.	KD	Vasorin KD reduced proliferation and induced the apoptosis of GSCs. In contrast, Vasorin KD in non-GSCs had little effect on cell viability.
Bordji et al. [52]	France (2014)	Lab (IV)	CL	U87, U251MG and GL15	HIF1α and HIF2α	class III beta-tubulin	HIF2α, not HIF1α, triggers bIII-t expression in hypoxic glioblastoma cells, facilitating tumor cell survival against DNA-targeting and tubulin-binding drugs, and promoting chemoresistance.	TF	HIF2α downregulation inhibits hypoxia-induced BIII-t expression in GL15 and U87 cells, enhancing glioblastoma cell sensitivity to chemotherapy.
Maurer et al. [53]	Germany (2019)	Lab (IV)	CL	LNT-229, U87, and T98G	HIF1α	TIGAR	TIGAR gene silencing enhances cell death associated with oxygen restriction.	KD	TIGAR KD enhances cell death under hypoxia and increases sensitivity to ionizing radiation, while also enhancing the effects of TMZ on cell density and clonogenicity.
Fan et al. [54]	USA (2014)	Lab (C)	CL	U251	HIF1α	Profilin-1	Pfn-1 phosphorylation drives endothelial angiocrine expression, promoting abnormal vascularization and glioblastoma progression via hypoxia-independent HIF1α induction.	KD	HIF1α KD disrupts the angiocrine feed-forward mechanism, normalizing vasculature.
Wei et al. [55]	China (2023)	Lab (C)	Mice and CL	U87, U251, and U373	HIF1α	Beclin-1	Beclin-1 suppression by 3-MA could reverse radioresistance induced by HIF1A under hypoxia.	KD	HIF1A KD improved glioblastoma radiosensitivity, and silencing Beclin-1 could reverse HIF1A-induced radioresistance under hypoxic conditions.
Coma et al. [56]	USA (2011)	Lab (IV)	CL	U87MG and A375SM	HIF1α	NRP2 and SEMA3F	SEMA3F inhibits tumor angiogenesis and metastasis. NRP2 is a receptor expressed by tumor cells that binds both SEMA3F and VEGF.	KD	The repression of NRP2 induced by DFO was hindered by HIF1α siRNA, validating that hypoxia-induced NRP2 repression is reliant on HIF1α.
Bao et al. [57]	USA (2018)	Lab (C)	Mice and CL	U251, U87, LN229, and HEK293FT	HIF1α	G9a and GLP	G9a/GLP-mediated K674 methylation decreases HIF1α transcriptional activity.	TF	G9a targets HIF1α, impairing tumorigenesis and glioblastoma cell migration by inhibiting its transcriptional activity and the expression of downstream targets like PTGS1, NDNF, SLC6A3, and Linc01132.
Lim et al. [58]	USA (2014)	Lab (C)	Mice and CL	HSR- glioblastoma 1 and JHH- glioblastoma 10	HIF1α	MCT4	MCT4 appears to regulate the proliferation, survival, and xenograft implantation/growth of some glioblastoma neurosphere lines.	KD	MCT4 KD reduces CD133+ cells and increases apoptosis, depleting glioblastoma stem-like cells and suppressing HIF transcription independently of lactate.
Lei et al. [59]	Taiwan (2023)	Lab (C)	Mice and CL	glioblastoma 8401, U251, glioblastoma04T, glioblastoma 09T, and HUVECs	HIF1α, HIF2α	GPx1	GPx1 is an antioxidant enzyme detoxifying H2O2 via the binding of HIF1α to GPx1 promoter. Exosomal GPx1 plays a critical role in providing resistance to oxidative stress and radiation.	KD	The inhibitors of GPx1 sensitize vascular endothelial cells to apoptosis triggered by oxidative stress or radiation, potentially restoring the sensitivity of tumor vessels to damage.
Joshi et al. [60]	California (2014)	Lab (IV)	CL	LN229-HRE-AP	HIF1α	MDM2 and PTEN-PI3K-AKT axis	HIF1α undergoes hypoxic degradation via the 26 S proteasome, facilitated by MDM2 as the E3 ligase. This process is regulated by the PTEN-PI3K-AKT signaling axis.	KD	The KD of PTEN in LN229-HRE-AP cells boosts HIF1α target gene transcription, while HIF1α degradation occurs under hypoxia.
Lulli et al. [61]	Italy (2020)	Lab (C)	Mice and CL	GSC, HNPC, and 293T	HIF1α	miR-370-3p	miR-370-3p functions as a tumor-suppressor, restraining glioma cell growth, migration, and invasion by targeting the lncRNAs NEAT1, HMGA2, and HIF1α.	KD	NEAT1 KD inhibited glioma cell proliferation, invasion, and migration.
Jung et al. [62]	USA (2019)	Lab (C)	Mice and CL	SCS from biopsy	HIF1α	NIX	NIX-mediated mitophagy regulates tumor survival in the hypoxic niche of the glioblastoma microenvironment.	KD	The KD of NIX dramatically reduced the expression of stem cell markers and self-renewal by suppressing the RHEB/AKT/HIF signaling cascade.
Jin et al. [63]	China (2022)	Lab (C)	Mice and CL	T98G, U87, U118, and U251	HIF1α	p21 (CDKN1A)	HIF1α binds to the p21 promoter’s HREs, boosting transcription; reciprocally, p21 enhances HIF1α mRNA transcription, sustaining its function during oxygen deficiency.	KD	The KD of HIF1A/p21 pathway inhibited glycolysis by downregulating Glut1 and LDHA and consequently caused the radiosensitivity of glioblastoma cells under hypoxic conditions.

HIF—hypoxia-inducible factor; Lab—laboratory study; C—combined design (in vivo and in vitro); IV—in vitro; IVEGF—vascular endothelial growth factor; PACAP—Pituitary Adenylate Cyclase-Activating Peptide; AMPK—AMP-activated Protein Kinase; ATM—Ataxia Telangiectasia Mutated; MIR210HG—microRNA 210 Host Gene; OCT1—Organic Cation Transporter 1; IGFBP2—Insulin-like Growth Factor Binding Protein 2; FGFR1—Fibroblast Growth Factor Receptor 1; Ror1—Receptor Tyrosine Kinase-Like Orphan Receptor 1; miR—microRNA; CA9—carbonic anhydrase 9; CXCR7—C-X-C Motif Chemokine Receptor 7; CXCR4 -C-X-C Motif Chemokine Receptor 4; IDH1—isocitrate dehydrogenase 1; SF/HGF—Scatter Factor/Hepatocyte Growth Factor; c-Met—Mesenchymal–Epithelial Transition Factor; S100A4—S100 Calcium Binding Protein A4; NMIIA—Non-Muscle Myosin IIA; NO—Nitric Oxide; BAG3—Bcl2 Associated Athanogene 3; HSP70—Heat Shock Protein 70; TDO2—Tryptophan 2,3-Dioxygenase; CK1α—casein kinase 1α; Foxp3—Forkhead Box P3; Tregs—Regulatory T Cells; ANPDD1A—Ankyrin Repeat And Death Domain Containing 1A; Nrf2—Nuclear Factor Erythroid 2-Related Factor 2; AEG-1—Astrocyte Elevated Gene 1; BMP2—Bone Morphogenetic Protein 2; Akt—Protein Kinase B; mTOR—Mammalian Target of Rapamycin; Akt/mTOR—Protein Kinase B/Mammalian Target of Rapamycin; N-Myc—Neuroblastoma Myc Proto-Oncogene; dsRNA—Double-stranded RNA; IAA—Iodoacetate; TDO2—Tryptophan 2,3-Dioxygenase; TGF-β1—Transforming Growth Factor Beta 1; E2F4—E2F transcription factor 4; Smad3—SMAD Family Member 3; TNF-α—Tumor Necrosis Factor Alpha; MHC-I—Major Histocompatibility Complex Class I; NF-κB—Nuclear Factor Kappa B; PI3K—Phosphoinositide 3-Kinase; ERK1/2—Extracellular Signal-Regulated Kinase 1/2; AHR—aryl hydrocarbon receptor; AP-1—activator protein 1; TNF—Tumor Necrosis Factor; DDX28—DEAD-Box Helicase 28; MBNL1—Muscleblind-Like Splicing Regulator 1; PTEN—Phosphatase and Tensin Homolog; PTEN-PI3K-AKT—Phosphatase and Tensin Homolog-Phosphoinositide 3-Kinase-Protein Kinase B; NRP2—Neuropilin 2; SEMA3F—Semaphorin 3F; G9a—G9a Histone Methyltransferase; GLP—G9a-Like Protein; MCT4—Monocarboxylate Transporter 4; GPx1—Glutathione Peroxidase 1; GPx1—Glutathione Peroxidase 1; CDKN1A—Cyclin-Dependent Kinase Inhibitor 1A. n/a—not available.

**Table 3:** Experimental investigations on hypoxia-inducible factors (HIFs) in glioblastoma models and animal subjects with induced tumors.

Reference	Country (Year)	Study Design	Species	Cell Line(s)	Targeted HIF	Related Factor	Role of HIF and Related Factors	Target/Systematic Therapy	Pharmacological Effects
Nardinocchi et al. [64]	Italy (2010)	Lab (C)	Mice and CL	U373	HIF1α	VEGF	The results of the luciferase assay showed that the hypoxia-induced as well as the cobalt-induced VEGF-luc activity was strongly inhibited by zinc.	Zinc	Zinc triggers HIF1α proteasomal degradation, potentially serving as a tumor progression inhibitor by suppressing pathways activated by VEGF, MDR1, and Bcl2 target genes, thereby enhancing anticancer therapies.
Maugeri et al. [65]	Italy (2021)	Lab (IV)	CL	U87	HIF1α	PACAP and PAC1R	HIF1α triggers angiogenic cascade via VEGF upregulation.	PACAP	PACAP inhibits VEGF release in the glioblastoma hypoxic microenvironment by reducing new vessel formation.
Ma et al. [66]	China (2022)	Lab (C)	Mice and CL	U251 and GL261	HIF1α	GLUT-1, GLUT-3, and HK2	The overexpression of HIF1α, GLUT-1, GLUT-3, and HK2 suggests HIF1α correlates with glucose metabolism in tumor tissue.	Acriflavine and PDT	PA group inhibited HIF1α expression and improved PDT efficacy in the treatment of recalcitrant glioblastoma.
D’Amico et al. [67]	Italy (2023)	Lab (IV)	Cell culture	U87 and A172	HIF1α	PACAP and VEGF	ADNP immunoreactivity was detected in most glial cells and its predominant expression in hypoxic areas overexpressing HIF1α.	The active fragment of ANDP—NAP.	ADNP modulated the HIF pathway by reducing VEGF secretion and migration.
D’Alessio et al. [68]	Italy (2016)	Lab (IV)	CL	U87, GCSCs, PCSCs, and HUVEC	HIF1α and HIF2α	VEGF, VEGFR1 and VEGFR2	Angiogenesis-related molecules	Anti-angiogenic therapy	The inhibition of neoangiogenetic events in glioblastoma.
Cristofaro et al. [69]	Italy (2020)	Lab (IV)	CL	Glioblastoma GSCs GB7	HIF1α	M2	M2 receptor activation by Ape is able to arrest cell proliferation in glioblastoma cell lines.	Ape/M2 agonists	Ape treatment in hypoxic conditions is able to inhibit cell cycle progression. It downregulates the expression of stemness markers and miR-210 levels.
Gagner et al. [70]	USA (2017)	Lab (C)	Mice and CL	CT-2A and GL261	HIF1α	CXCR4 and POL5551	POL5551 inhibits CXCR4 binding to its ligand, SDF-1α, and reduces hypoxia- and stromal cell-derived factor-1a-mediated migration dose-dependently.	B20-4.1.1 and POL5551	When combined with B20-4.1.1, POL5551 reduced glioma invasion and the number of tumor-associated MGCs, which promote glioma growth and dissemination.
Lin et al. [71]	China (2024)	Lab (C)	Mice and CL	C6 and U251	HIF1α	n/a	The expression level of HIF1α is closely related to tumor cell proliferation, differentiation, apoptosis, phenotype determination, angiogenesis, energy metabolism, and resistance to therapy.	Borneol and TMZ	Borneol has the potential to enhance the sensitivity of TMZ chemotherapy, with HIF1α being a promising target for enhancing the antitumor effectiveness of TMZ. This association is closely linked to the facilitation of the autophagic degradation of HIF1α.
Douglas et al. [72]	USA (2023)	Lab (C)	Mice and CL	U251, D-54MG, U87MG, and CHLA-200. GSC: DB70, DB76, DB77, and DB81, 192, and 83MES	HIF1α	LonP1 and CT-L	LonP1, an ATP-dependent protease, is directly upregulated by HIF1α, with increased expression and CT-L proteasome activities observed in gliomas, correlating with high tumor grade and poor patient survival.	BT317	BT317 has a dual LonP1 and CT-L inhibition profile and induces increased ROS production and autophagy-dependent cell death in clinically relevant, IDH mutant malignant astrocytoma.
Arienti et al. [73]	Italy (2021)	Lab (IV)	CL	G34, G40, G44, and CHME-5	HIF1α	n/a	The expression of HIF1α stimulates the upregulation of the glycolysis metabolic pathway, boosting ATP production necessary for cell survival and proliferation.	HBO	HBO inhibits cell proliferation, downregulates HIF1α expression, and induces glucose metabolism reprogramming.
Lin et al. [74]	USA (2015)	Lab (C)	Mice and CL	U87 and LN229	HIF1α	IGFBP2 and IGFI	The activation of IGFIR by IGFI and subsequent downstream signaling lead to malignant cell proliferation, motility, and metastasis.	GFBP2-HIF1α targeting	Blocking specific molecular interactions within the insulin signaling pathway could potentially result in a notable decrease in glioblastoma growth.
Lund et al. [75]	Denmark (2004)	Lab (IV)	CL	U87	HIF1α	VEGF and angiopoetin-1, -2, -4	VEGF protects endothelial cells from apoptosis via Raf activation, while Ang-1 and Ang-2 are essential for angiogenesis, and Ang-4 induces Tie-2 receptor autophosphorylation.	IR	The combinations of radiation therapy and therapy targeting the signaling pathways of VEGF have proven more effective than irradiation alone in animal models.
Hofstetter et al. [76]	USA (2012)	Lab (IV)	Cell culture	TSCs (334, 974, and 980)	HIF1α	PP2A	Hypoxia-induced PP2A halts cell proliferation, decreasing metabolic activity, and promotes survival of TSCs in severe hypoxia.	The modulation of PP2A	Possible synergistic effects of chemotherapy with PP2A inhibition.
Bi et al. [77]	China (2021)	Lab (IV)	CL	U251	HIF1α	ARA1 and ARA3	PSH decreases HIF1α expression via ARA3 inactivation and induces cell cycle arrest via ARA1.	PSH	PSH reduced U251 cell viability via the inhibition of ARA1 and ARA3 expression and further inhibited Akt and 44/42 MAPK phosphorylation, induced apoptosis, and cell cycle arrest.
Ma et al. [66]	China (2022)	Lab (C)	Mice and CL	U251 and GL261	HIF1α	GLUT1, GLUT3, and HK2	Human glioblastoma tissues showed extensive overexpression of HIF1α, GLUT-1, GLUT-3, and HK2, suggesting HIF1α correlated with glucose metabolism in tumor tissue.	PDT and acriflavine	Acriflavine combined with PDT attenuated the expression of HIF1α, GLUT-1, GLUT-3, and HK2 and improved tumor suppression.
Khoei et al. [78]	Iran (2016)	Lab (IV)	Cell culture	U87	HIF1α	n/a	Hypoxia activates the HIF1α pathway and reduces the sensitivity of tumor cells to radiation and chemotherapeutic drugs.	Res, MX, and IUdR	A combination of MX and Res with IUdR can decrease colony formation ability and increase DNA damage of gamma-ray radiation in 350 mm spheroids. The cytotoxic effect of Rad and therapeutic ratio increases.
Liu et al. [79]	China (2020)	Lab (C)	Mice and CL	G422-Glioblastoma	HIF1α	n/a	HIF1α is a mediator in the mechanism of chemotherapy resistance.	RT/TMZ supplemented with mannose	RT/TMZ/Man could offer a disease cure for glioblastoma through metabolically abolishing the HIF-1-mediated resistance.
Dačević et al. [80]	Serbia (2013)	Lab (IV)	CL	U87, U87-TxR, NCI-H460, NCI-H460/R, and HaCaT	HIF1α	Pgp, VEGF, and GSH	P-gp activity governs MDR development. GSH is implicated in detoxification and VEGF has a role in tumor angiogenesis and progression.	SF	SF hampers the growth of cancer cells by integrating its phosphorylated derivatives into DNA. Moreover, SF diminishes the levels of HIF1α, which governs the expression of both P-gp and VEGF. As a consequence, SF’s influence on multidrug resistance (MDR) stems from its ability to inhibit the GSH detoxification system.
Ishii et al. [81]	Japan (2016)	Lab (IV)	CL	T98G	HIF1α and HIF2α	SOX2 and NANOG	SOX2 and NANOG, transcription factors crucial for embryonic stem cell self-renewal and pluripotency, also play critical roles in glioblastoma tumorigenesis.	The targeting of the peri-necrotic niche	Eradicating glioblastoma cells and overcoming the therapeutic resistance of glioblastomas.
Li et al. [82]	China (2023)	Lab (C)	Mice and CL	U251 and U87	HIF1α	GLUT1	The HIF-1/GLUT-1 axis enhanced the cytotoxicity of temozolomide in gliomas as a result of PDT treatment, which was influenced by ROS.	TMZ and PDT	Photodynamic therapy boosts the cytotoxic effects of temozolomide on glioblastoma by reshaping anaerobic glycolysis.
Bernstock et al. [83]	USA (2017)	Lab (IV)	CL	U251, LN229, Mz18, and SH-SY5Y	HIF1α	SUMO	SUMO maintains cellular function under conditions of stress.	Topotecan	Topotecan reduces the levels of global SUMO conjugation, CDK6, and HIF1α in glioblastoma cells, thereby affecting both the cell cycle and metabolic profile.
Tafani et al. [84]	Italy (2011)	Lab (C)	Mice and CL	Biopsy	HIF1α	HK2 and VEGF	After 4 h of hypoxia, there was an elevation in mRNA expression for HIF1α. VEGF mRNA demonstrated an increase during hypoxia treatment, while HK2 mRNA exhibited increases after 4, 24, and 48 h of hypoxia.	Digoxin and acriflavine	The prevention of HIF1α protein synthesis and dimerization.
Muh et al. [85]	USA (2014)	Lab (IV)	Mice	U87 and U373	HIF1α	PTEN-PI3K	This synergistic activity was correlated with a synergistic suppression of HIF1α accumulation under hypoxic conditions in glioma models.	LY294002 and 2ME2	Drugs demonstrated synergy in blocking HIF1α accumulation in glioblastoma cell lines.
Pore et al. [86]	United States (2006)	Lab (C)	Mice and CL	U87 and U251	HIF1α	PI3K/Akt	Nelfinavir downregulates VEGF and HIF-1 expression through the inactivation of PI3K/Akt pathways.	Nelfinavir and amprenavir	Nelfinavir downregulates VEGF and HIF-1 expression through the inactivation of PI3K/Akt pathways, decreases angiogenesis in vivo, and downregulates HIF1α through the inhibition of protein synthesis. Amprenavir inhibits VEGF and HIF-1 expression in glioblastoma cells but not in normal human astrocytes.
Sugimoto et al. [87]	Japan (2017)	Lab (IV)	CL	U87	n/d	GFAP and CBR1	Hypoxia decreases the expression of CBR1 and glial fibrillary acidic protein while increasing the expression of VEGF and cyclooxygenase-2.	WIN 55,212-2	CB engagement induces cell death in U-87 MG cells under normoxic conditions, with CB agonist-induced death being reduced in hypoxic conditions.
Lin et al. [88]	Taiwan (2021)	Lab (IV)	CL	U251	HIF1α	PPARα	Hypoxia-induced HIF1α regulates pH-regulating proteins in glioblastoma.	Fenofibrate	Fenofibrate effectively inhibits hypoxia-induced HIF1α and CA9 expression in glioblastoma by activating HO-1 via AMPK and promoting HIF1α degradation, suggesting its potential as a multi-pathway anti-glioblastoma agent.

HIF—hypoxia-inducible factor; Lab—laboratory study; C—combined design (in vivo and in vitro); IV—in vitro; VEGF—vascular endothelial growth factor; PACAP—Pituitary Adenylate Cyclase-Activating Polypeptide; GLUT—glucose transporter; HK—hexokinase; ACF—acriflavine; PA—photodynamic therapy; ADNP—Activity-Dependent Neuroprotective Protein; ANDP—Activity-Dependent Neurotrophic Factor-Derived Peptide; MDR—multidrug resistance; MDR1—multidrug resistance protein 1; Bcl2—B-cell lymphoma 2; GSCs—glioma stem cells; Ape—arecaidine propargyl ester; MGCs—Multinucleated Giant Cells; CXCR4—C-X-C Chemokine Receptor Type 4; SDF-1α—stromal cell-derived factor 1 alpha; POL5551—CXCR4 antagonist; HBO—Hyperbaric Oxygen; IGF—Insulin-like Growth Factor; IGFBP—Insulin-like Growth Factor Binding Protein; SOX2—Sex-determining region Y-box 2; NANOG—Homeobox Protein Nanog; TSCs—tumor stem cells; PP2A—Protein Phosphatase 2A; PSH—Paris saponin H; ARA—Androgen Receptor Antagonist; ROS—reactive oxygen species; TMZ—temozolomide; SUMO—Small Ubiquitin-like Modifier; CDK6—Cyclin-Dependent Kinase 6; PDT—photodynamic therapy; PTEN—Phosphatase and Tensin Homolog; PI3K—Phosphoinositide 3-Kinase; Akt—Protein Kinase B; 2ME2—2-Methoxyestradiol; GFAP—glial fibrillary acidic protein; PPARα—Peroxisome Proliferator-Activated Receptor Alpha; CA9—carbonic anhydrase 9; CB—cannabinoid receptor; AMPK—AMP-activated Protein Kinase; HO-1—Heme Oxygenase 1; BT317—LonP1 and CT-L proteasome inhibition; Res—resveratrol; MX—methoxyamine; IUdR—iododeoxyuridine; CBR1—cannabinoid receptor 1. n/a—not available.

**Table 4:** Experimental investigations on hypoxia-inducible factors (HIFs) in glioblastoma models and animal subjects with induced tumors with combined genetic and targeted/systematic therapy.

Reference	Country (Year)	Study Design	Species	Cell Line(s)	Targeted HIF	Related Factor	Role of HIF and Related Factors	Gene Modification	Effect of Gene Modification	Targeted Therapy	Pharmacological Effects
Huang et al. [90]	China (2018)	Lab (IV)	CL	U87	HIF1α	PI3K/Akt/mTOR	PI3K/Akt/mTOR/HIF1α pathway is involved in enhancing the migration and invasion of human glioblastoma U87 cells under hypoxia.	TF	The enhancements of the migration and invasion of U87 cells under hypoxia could be suppressed by the mTOR pathway siRNA by targeting HIF1α.	2-ME, LY294002, rapamycin, and p70S6K siRNA	2-ME is an HIF1α inhibitor that reduces the migration and invasion of glioblastoma cells. The inhibitors of PI3K/Akt/mTOR, LY294002, and rapamycin, reduced the migration, invasion, and HIF1α protein expression. p70S6K siRNA suppressed the migration, invasion, and HIF1α expression under hypoxia.
Chhipa et al. [91]	USA (2018)	Lab (C)	Mice and CL	U87, A172, T98G, and HEK 293T	HIF1α	AMPK (AMPK/CREB1 axis)	By phosphorylating CREB1, AMPK enhances HIF1α and GABPA transcription to support glioblastoma bioenergetics.	KD and KO	Silencing CREB1 decreases HIF1α activity, cell viability, and GSC bioenergetics, while the knockout of AMPKα1 enhances glycolysis and accelerates tumorigenesis.	Bafilomycin	AMPK inhibition reduces GSC viability and has antitumorigenic effects.
Pang et al. [92]	USA (2023)	Lab (C)	Mice and CL	293T	HIF1α	LGMN	LGMN is specifically expressed in TAMs and regulated by HIF1α	KD and KO	BMDMs from HIF1α-mKO mice exhibited aberrantly diminished Lgmn expression levels, while Lgmn-mKD mice displayed a marked extension in survival compared to control mice.	Anti-PD1	The blockade of the HIF1α-LGMN axis synergizes with anti-PD1 therapy in glioblastoma.
Hu et al. [93]	USA (2012)	Lab (C)	Mice and CL	U87, T98G, U251, U138, A172, G55, SF8244, SF8557, and U373	HIF1α	HIF1α/AMPK	HIF1α and AMPK control hypoxia-induced LC3 changes, while BNIP3 expression depends solely on HIF1α, and p62 degradation occurs independently of both.	KO and TF	The knockdown of the essential autophagy gene ATG7 promotes bevacizumab responsiveness.	BEV and chloroquine	BEV treatment increased BNIP3 expression and hypoxia-driven growth in glioblastoma xenografts, reversed by chloroquine, an autophagy inhibitor.
Chou et al. [94]	Taiwan (2012)	Lab (C)	Mice and CL	U87, glioblastoma 8401, and U251	HIF1α	ABCB1	Cycling hypoxic stress increases chemoresistance via HIF–1-mediated ABCB1 induction.	KD	When the induction of ABCB1 was inhibited by siRNA, the chemotherapy resistance induced by cycling hypoxic stress decreased.	YC-1	YC-1 combined with BCNU chemotherapy decreased ABCB1 induction and made therapy more effective.
Barliya et al. [95]	Israel (2011)	Lab (IV)	CL	ARPE-19, U87, and RCC-C2VHL−/−	HIF1α	hsp90	Hsp90 mediates the pathways vital for angiogenesis, cell migration, and invasion.	TF	Hypericin interferes with VEGF promoter activation in tumor cell lines.	Hypericin	The hypericin-induced degradation of hsp90 client proteins compromises the pathways involved in angiogenesis, cell migration, and invasion.
Hsieh et al. [96]	Taiwan (2011)	Lab (C)	Mice and CL	glioblastoma 8401 and U87	HIF-1	NADPH oxidase subunit 4-mediated reactive oxygen species	Cycling hypoxic stress significantly increases ROS production, HIF-1 activation, and tumor growth. Nox4 is a critical mediator of these processes.	KD	Blocking ROS production through Nox4 shRNA inhibits tumor growth induced by cycling hypoxia or the tumor microenvironment.	Tempol	Tempol treatment inhibits tumor growth induced by cycling hypoxia or the tumor microenvironment.
Kannappan et al. [97]	United Kingdom (2022)	Lab (C)	Mice and CL	U87MG, U251MG, and U373MG	HIF1α and HIF2α	NF-kB	NF-kB, HIF1α, and HIF2α induce the expression of key EMT- and metastasis-related genes and promote glioblastoma cell migration and invasion.	TF	The expression of HIF2α mRNA was upregulated by HIF1α transfection but not vice versa.	Disulfiram	Disulfiram inhibits NF-kB activity and targets hypoxia-induced GSCs. It shows selective toxicity to glioblastoma cells, eradicates GSCs, and blocks migration and invasion.
Joseph et al. [98]	The Netherlands (2015)	Lab (IV)	CL	U87, SNB75, and U251	HIF1α and HIF2α	ZEB1 (HIF1α-ZEB1 axis)	HIF1α–ZEB1 signaling axis promotes hypoxia-induced mesenchymal shift and invasion in glioblastoma in a cell line-dependent fashion.	KD	The ShRNA-mediated knockdown of HIF1α, and not HIF2α, prevented hypoxia-induced mesenchymal transition.	Digoxin	Digoxin inhibits HIF1α mRNA translation.
Caragher et al. [99]	USA (2019)	Lab (C)	Mice and CL	U251, glioblastoma 43, glioblastoma 12, glioblastoma 5, glioblastoma 6, and glioblastoma 39	HIF1α and HIF2α	DRD2	The activation of DRD2 triggers the expression of HIF proteins and enhances the capacity for sphere formation, which serves as an indicator of the GIC state and tumorigenicity.	KD	The SH-RNA-mediated knockdown of DRD2 showed a significant reduction in sphere-forming capacity.	Chlorpromazine	The inhibition of glioblastoma growth by blocking the dopamine signaling pathway.
Peng et al. [100]	China (2021)	Lab (C)	Mice and CL	U251	HIF1α	PDGFD-PDGFRα	Under normoxic or mild-hypoxic conditions, HIF1α binds to the PDGFD proximal promoter and PDGFRA intron enhancers in glioblastoma cells, leading to the induction of their expression.	KD and KO	PDFGRA knockdown extends the survival of xenograft mice, inhibits cell growth and invasion in vitro, and eradicates tumor growth in vivo.	Echinomycin	Echinomycin induces glioblastoma cell apoptosis and effectively inhibits the growth of glioblastoma in vivo by simultaneously targeting the HIF1α-PDGFD/PDGFRα-AKT feedforward pathway.
Han et al. [101]	China (2015)	Lab (C)	Mice and CL	U87 and U251	HIF1α	NF-κB/RelA-PKM2	NF-κB/RelA is involved in proliferation, anti-apoptosis, angiogenesis, and metastasis, promoting aerobic glycolysis via the transcriptional activation of PKM2.	TF	NF-κB/RelA promotes glioblastoma cell glycolysis depending on PKM2.	Fenofibrate	FF inhibits glioblastoma glycolysis in a dose-related manner depending on PPARα activation. It inhibits the transcriptional activity of NF-κB/RelA and disrupts its association with HIF1α.
Dominguez et al. [102]	USA (2013)	Lab (C)	Mice and CL	U251, U87, A375, MDA-MB-231, HeLa, and human fibroblast cell lines	HIF1α	DGKα	DGKα and its product, phosphatidic acid, are associated with multiple oncogenic pathways such as mTOR, HIF1α, and Akt.	KD	In cancer cells, the inhibition of DGKα results in cell toxicity through caspase-mediated apoptosis. The reduced expression of mTOR and HIF1α significantly contributes to the cytotoxic effects observed upon DGKα knockdown and inhibition in cancer.	R50922 and R59949	Induced caspase-mediated apoptosis in glioblastoma cells and in other cancers, but lacked toxicity in non-cancerous cells.
Hsieh et al. [103]	Taiwan (2015)	Lab (C)	Mice and CL	U251, U87, and glioblastoma 8401	HIF1α and HIF2α	Livin proteins	HIF1α regulates Livin transcription in hypoxia, promoting anti-apoptosis in glioblastoma and enhancing radioresistance and chemoresistance.	KD	The knockdown of Livin suppresses tumor hypoxia-induced TR and generates a synergistic suppression of antitumor growth and tumor cell death.	Cell-permeable peptide TAT-Lp15	Livin blockage enhances the efficiency of radiation plus temozolomide treatment in glioblastoma xenografts.
Ahmed et al. [104]	UK (2018)	Lab (IV)	CL	U251, U87, and SNB219	HIF1α and HIF2α	CD133	CD133 is a cell surface marker used to identify glioblastoma cancer stem cells.	KD	HIF1α and HIF2α knockdown led to a reduced CD133 expression. CD133 knockdown increases the sensitivity of glioblastoma cells to cisplatin.	Cisplatin	The hypoxia-induced cisplatin sensitivity of glioblastoma cells may be HIF-independent and may be directly or indirectly induced via CD133 activation.
Lee et al. [105]	Korea (2017)	Lab (C)	Mice and CL	Biopsy	HIF1α	ERK1/2 and VEGF	ERK1/2 signaling and VEGF, a HIF1α downstream target, contribute to solid tumor pathogenesis.	TF	DT at clinically relevant concentrations reduces hypoxia-induced HIF1α protein accumulation and downstream signaling pathways.	Digitoxin	DT at clinically achievable concentration functions as an inhibitor of HIF1α.
Bar et al. [106]	USA (2010)	Lab (C)	Mice and CL	HSR-glioblastoma 1 and HSR-glioblastoma 2	HIF1α	CD133	HIF1α induces CD133 expression and enhances the stem-like tumor subpopulation in hypoxia.	TF	An elevated percentage of CD133 positive cells.	Digoxin	Digoxin suppressed HIF1α protein expression, HIF1α downstream targets, and slowed tumor growth.
Chen et al. [107]	China (2015)	Lab (C)	Mice and CL	U251, U87, and glioblastoma 8401	HIF1α	NF-κB and Bc-xl	Cycling hypoxia mediates Bcl-xL expression via HIF1α or NF-κB activation, which results in chemoresistance.	KD	Bcl-xL knockdown inhibited cycling hypoxia-induced chemoresistance.	Tempol, YC-1, and Bay 11-7082	The suppression of the cycling hypoxia-mediated Bcl-xL induction.
Li et al. [108]	India (2020)	Lab (C)	Mice and CL	U87 and U251	HIF1α	IDH1-R132H	The overexpression of IDH1-R132H increased the expression of HIF1α and the downregulation of HIF1α suppressed the IDH1-R132H-induced effect on glioblastoma.	KD	The KD of FAT1 inhibited the IDH1-R132H-induced reduction in tumor growth in xenograft mice.	TMZ	The overexpression of IDH1-R132H led to reduced cell proliferation, increased apoptosis, decreased migration and invasion, enhanced TMZ-induced cytotoxicity, and diminished tumor growth in xenograft mice.
Ge et al. [109]	China (2018)	Lab (C)	Mice and CL	U87MG and HEK293T	HIF1α	miR-26a	HIF1α/miR-26a axis strengthens the acquisition of TMZ resistance through the prevention of Bax and Bad in mitochondria dysfunction in glioblastoma.	TF	HIF1α serves as a pivotal upstream regulator of miR-26a expression in glioma.	TMZ	miR-26a is an important regulator of TMZ resistance induced by hypoxia, which can effectively protect mitochondria function and reduce apoptosis by targeting bax and bad.
Liao et al. [110]	China (2022)	Lab (C)	Mice and CL	U251, U87, A172, GSC11, GSC20, GSC262, GSC267, GSC295, GSC28, GSC284, and GSC627	HIF1α	PRMT3	PRMT3 promotes glioblastoma progression by enhancing HIF1α-mediated glycolysis and metabolic rewiring.	KD	The reduced proliferation and migration of glioblastoma cell lines and patient-derived GSC in cell culture and inhibited tumor growth.	SGC707	The targeting of PRMT3 decreases HIF1α expression and glycolytic rates in glioblastoma cells and inhibits glioblastoma growth.
Kioi et al. [111]	California (2010)	Lab (C)	Mice and CL	U251 and U87	HIF1α	SDF-1/CXCR4	BMDCs are recruited to tumors through the HIF-1-dependent interaction of SDF-1 and its receptor, CXCR4.	TD	AMD3100 enhanced the radiosensitivity.	AMD3100	AMD3100 is an inhibitor of SDF-1/CXCR4 interactions, which blocks the vasculogenesis pathway.
Boso et al. [112]	Italy (2019)	Lab (IV)	CL	Biopsy	HIF1α	β-catenin/TCF1	In hypoxic glioblastoma cells, the β-catenin/TCF1 complex recruits HIF1α to promote the transcription of genes associated with neuronal differentiation.	TF	Cells silenced for TCF1 experienced a complete inhibition of their neuronal differentiation potential.	TCF4E	TCF4E possesses inhibitory effects on gene transcription.

CL—cell line; Lab—laboratory study; C—combined design (in vivo and in vitro); IV—in vitro; KD—knockdown; KO—knockout; TF—transfection; TD—transduction; siRNA—small interfering RNA; PDGFD—platelet-derived growth factor D; PDGFRα—platelet-derived growth factor receptor alpha; AMPK—AMP-activated Protein Kinase; CREB1—cAMP Response Element-Binding Protein 1; GABPA—GA Binding Protein transcription factor subunit Alpha; LGMN—Legumain; TAMs—Tumor-Associated Macrophages; mKO—Myeloid Cell-Specific knockout; BMDMs—Bone Marrow-Derived Macrophages; Nox4—NADPH Oxidase 4; ROS—reactive oxygen species; LC3—Microtubule-Associated Protein 1A/1B-Light Chain 3; BNIP3—Bcl2/adenovirus E1B 19kDa Interacting Protein 3; ATG7—Autophagy-Related 7; NF-kB—Nuclear Factor Kappa B; EMT—Epithelial–Mesenchymal Transition; GIC—glioma-initiating cells; DRD2—Dopamine Receptor D2; DGKα—Diacylglycerol Kinase Alpha; ERK1/2—Extracellular Signal-Regulated Kinase 1/2; VEGF—vascular endothelial growth factor; PKM2—Pyruvate Kinase M2; PKM2—Pyruvate Kinase M2; DGKα—Diacylglycerol Kinase Alpha; TR—Tumor Regrowth; CD133—Prominin-1; ZEB1—Zinc Finger E-Box Binding Homeobox 1; NF-κB—Nuclear Factor Kappa B; Bcl-xL—B-cell lymphoma-extra-large; IDH1—isocitrate dehydrogenase 1; FAT1—FAT Atypical Cadherin 1; TMZ—temozolomide; PRMT3—Protein Arginine Methyltransferase 3; SDF-1—stromal cell-derived factor 1; CXCR4—C-X-C Motif Chemokine Receptor 4; TCF1—transcription factor 1; TCF4E—transcription factor 4E; AMD3100—Plerixafor; GIC—glioma-initiating cell.

**Table 5:** Included clinical studies.

Reference	Country (Year)	Study Design	Sample (N)	Age	Gender (Male/Female)	Target(s) (Type of HIF)	Findings
Chen et al. [114]	China (2019)	Prospective	42	26–76	17/25	CAV1 and HIF1α	HIF1α is more expressed in the nucleus and cytoplasm of neoplastic cells. HIF1α correlated with high CAV1 expression, larger glioblastoma size, and lesser survival time.
Bache et al. [115]	Germany (2015)	Retrospective	41	Median: 63	16/18	HIF1α, HIF2α, CA9, VEGF, GLUT-1, OPN, survivin, EGFR, hTERT, and OCT4	HIF2α, CA9, VEGF, hTERT, and OCT4 were higher in glioblastoma than in tumor-free brain tissues; the mRNA expression levels of HIF genes resulted in shorter survival times for patients with glioblastoma; the mRNA expression levels of HIF and stem cell-associated genes are important glioblastoma markers.
Erpolat et al. [116]	Turkey (2012)	Retrospective	79	Median: 49	n/d	HIF1α, CA9, and OPN	High levels of cytoplasmic and nuclear HIF1α, CA9, and osteopontin correlated with shorter survival, especially with high hypoxic scores, with high hypoxic score-1 being the main independent negative predictor for survival.
Clara et al. [122]	Brazil (2014)	Retrospective	208	Median: 56	127/81	HIF1α	HIF1α expression in glioblastoma is correlated with increased vascular density and with VEGF and PDGF-C expression. Nuclear HIF1α and VEGF staining also correlated with survival.
Kaynar et al. [117]	Turkey (2008)	Prospective	26	Median: 51	17/9	HIF1α	HIF1α levels were elevated in glioblastoma, indicating a role in angiogenesis possibly beyond hypoxia.
El-Benhawy et al. [123]	Egypt (2022)	Prospective	80	Mean: 49.49	58/22	HIF1α, VEGF, OPN, erythropoietin, caveolin-1, GLUT-1, and LDH	Serum hypoxia biomarkers, including HIF1α, VEGF, and LDH, increased significantly after radiotherapy in patients with glioblastoma, indicating their potential role in tumor progression and treatment response.
Nobuyuki et al. [118]	Japan (2004)	Prospective	60	Median: 58.7	33/27	HIF1α	HIF1 serves as a hypoxic sensor in tumors like glioblastoma, with its expression level indicating radioresistance and guiding postoperative radiotherapy protocols.
Ji et al. [119]	China (2013)	Prospective	68	Mean: 48	46/22	HIF1α	High HIF1α expression in glioblastoma correlates with poorer outcomes, including shorter overall and progression-free survival, suggesting its potential as a marker for targeted treatment.
Sfifou et al. [120]	Morocco (2021)	Prospective	22	Mean: 54	n/d	HIF1α	Patients with negative HIF1α expression and positive IDH1 expression have a better prognosis, with statistically significant differences observed in overall survival rates, indicating HIF1α as a potential prognostic marker.
Potharaju et al. [121]	India (2019)	Prospective	87	Median: 55	59/28	HIF1α	The strong nuclear staining of HIF1α was observed in 48% of the samples, correlating with poor prognosis independently. Patients with strong HIF1α and TERT expression had the worst prognosis, indicating HIF1α as a potential prognostic marker in glioblastoma.

n/d—not disclosed; CAV1—caveolin-1; HIF1α—hypoxia-inducible factor 1-alpha; HIF2α—hypoxia-inducible factor 2-alpha; CA9—carbonic anhydrase 9; VEGF—vascular endothelial growth factor; GLUT-1—glucose transporter 1; OPN—osteopontin; EGFR—Epidermal Growth Factor Receptor; hTERT—Human Telomerase Reverse Transcriptase; OCT4—Octamer-binding Transcription Factor 4; LDH—Lactate Dehydrogenase; IDH1—isocitrate dehydrogenase 1.

## Data Availability

Data are contained within the article.

## Appendix A

**Search**	**(Glioblastoma) AND (Hypoxia-Inducible Factors)**
Base: MEDLINE (PubMed)	
Filter	None
Search query	(“glioblastoma”[MeSH Terms] OR “glioblastoma”[All Fields] OR “glioblastomas”[All Fields]) AND ((“hypoxia”[MeSH Terms] OR “hypoxia”[All Fields] OR “hypoxia s”[All Fields] OR “hypoxias”[All Fields]) AND (“induce”[All Fields] OR “induced”[All Fields] OR “inducer”[All Fields] OR “inducers”[All Fields] OR “induces”[All Fields] OR “inducibilities”[All Fields] OR “inducibility”[All Fields] OR “inducible”[All Fields] OR “inducing”[All Fields]) AND (“factor”[All Fields] OR “factor s”[All Fields] OR “factors”[All Fields]))
Results	558 papers
Base: Web of Science	
Filter	None
Search query	TS = (“glioblastoma” OR “glioblastomas”) AND TS = (“hypoxia inducible factors” OR “hypoxia-inducible factors” OR “HIFs”)
Results	89 papers
Base: Scopus	
Filter	None
Search query	TITLE-ABS-KEY(“glioblastoma”) AND TITLE-ABS-KEY(“hypoxia inducible factors” OR “HIFs”)
Results	671 papers

## Appendix B

**Section/Topic**	**#**	**Checklist Item**	**Reported on Page #**
Title			
Title	1	Identify the report as a systematic review, meta-analysis, or both.	1
Abstract			
Structured summary	2	Provide a structured summary including, as applicable: background; objectives; data sources; study eligibility criteria, participants, and interventions; study appraisal and synthesis methods; results; limitations; conclusions and implications of key findings; and systematic review registration number.	1
Introduction			
Rationale	3	Describe the rationale for the review in the context of what is already known.	2
Objectives	4	Provide an explicit statement of the questions being addressed with reference to the participants, interventions, comparisons, outcomes, and study design (PICOS).	2
Methods			
Protocol and registration	5	Indicate if a review protocol exists, if and where it can be accessed (e.g., Web address), and, if available, provide registration information including registration number.	2
Eligibility criteria	6	Specify study characteristics (e.g., PICOS and length of follow-up) and report characteristics (e.g., years considered, language and publication status) used as criteria for eligibility, giving rationale.	3
Information sources	7	Describe all information sources (e.g., databases with dates of coverage and contact with study authors to identify additional studies) in the search and date last searched.	3
Search	8	Present a full electronic search strategy for at least one database, including any limits used, such that it could be repeated.	Appendix A
Study selection	9	State the process for selecting studies (i.e., screening, eligibility, included in the systematic review, and, if applicable, included in the meta-analysis).	3
Data collection process	10	Describe the method of data extraction from the reports (e.g., piloted forms, independently and in duplicate) and any processes for obtaining and confirming data from the investigators.	3
Data items	11	List and define all variables for which data were sought (e.g., PICOS and funding sources) and any assumptions and simplifications made.	3
Risk of bias in individual studies	12	Describe the methods used for assessing the risk of bias in individual studies (including the specification of whether this was performed at the study or outcome level), and how this information is to be used in any data synthesis.	N/A
Summary measures	13	State the principal summary measures (e.g., risk ratio and the difference in means).	N/A
Synthesis of results	14	Describe the methods of handling data and combining the results of studies, if performed, including the measures of consistency (e.g., I^2^) for each meta-analysis.	N/A
Risk of bias across studies	15	Specify any assessment of the risk of bias that may affect the cumulative evidence (e.g., publication bias and selective reporting within studies).	N/A
Additional analyses	16	Describe the methods of additional analyses (e.g., sensitivity or subgroup analyses and meta-regression), if performed, indicating which were pre-specified.	N/A
Results			
Study selection	17	Give the number of studies screened, assessed for eligibility, and included in the review, with reasons for exclusions at each stage, ideally with a flow diagram.	Figure 1
Study characteristics	18	For each study, present characteristics for which data were extracted (e.g., study size, PICOS, and follow-up period) and provide the citations.	3
Risk of bias within studies	19	Present data on the risk of bias of each study and, if available, any outcome level assessment (see item 12).	N/A
Results of individual studies	20	For all outcomes considered (benefits or harms), present, for each study: (a) simple summary data for each intervention group and (b) effect estimates and confidence intervals, ideally with a forest plot.	Table 1, Table 2 and Table 3
Synthesis of results	21	Present the results of each meta-analysis performed, including confidence intervals and the measures of consistency.	N/A
Risk of bias across studies	22	Present the results of any assessment of the risk of bias across studies (see item 15).	N/A
Additional analysis	23	Give the results of additional analyses, if performed (e.g., sensitivity or subgroup analyses and meta-regression [see item 16]).	N/A
Discussion			
Summary of evidence	24	Summarize the main findings including the strength of evidence for each main outcome; consider their relevance to key groups (e.g., healthcare providers, users, and policy makers).	4–8
Limitations	25	Discuss limitations at the study and outcome level (e.g., risk of bias), and at the review level (e.g., the incomplete retrieval of identified research and reporting bias).	10
Conclusions	26	Provide a general interpretation of the results in the context of other evidence, and implications for future research.	11
Funding			
Funding	27	Describe the sources of funding for the systematic review and other support (e.g., supply of data); and the role of funders for the systematic review.	11
N/A—Not Available			

## References

[1] Shah S. (2024). Novel Therapies in Glioblastoma Treatment: Review of Glioblastoma; Current Treatment Options; and Novel Oncolytic Viral Therapies. Med. Sci..

[2] Begagić E., Bečulić H., Đuzić N., Džidić-Krivić A., Pugonja R., Muharemović A., Jaganjac B., Salković N., Sefo H., Pojskić M. (2024). CRISPR/Cas9-Mediated Gene Therapy for Glioblastoma: A Scoping Review. Biomedicines.

[3] Fuchs Q., Pierrevelcin M., Messe M., Lhermitte B., Blandin A.F., Papin C., Coca A., Dontenwill M., Entz-Werlé N. (2020). Hypoxia Inducible Factors’ Signaling in Pediatric High-Grade Gliomas: Role, Modelization and Innovative Targeted Approaches. Cancers.

[4] Kaur B., Khwaja F.W., Severson E.A., Matheny S.L., Brat D.J., Van Meir E.G. (2005). Hypoxia and the hypoxia-inducible-factor pathway in glioma growth and angiogenesis. Neuro Oncol..

[5] Renfrow J.J., Soike M.H., Debinski W., Ramkissoon S.H., Mott R.T., Frenkel M.B., Sarkaria J.N., Lesser G.J., Strowd R.E. (2018). Hypoxia-inducible factor 2α: A novel target in gliomas. Future Med. Chem..

[6] Jensen R.L., Mumert M.L., Gillespie D.L., Kinney A.Y., Schabel M.C., Salzman K.L. (2014). Preoperative dynamic contrast-enhanced MRI correlates with molecular markers of hypoxia and vascularity in specific areas of intratumoral microenvironment and is predictive of patient outcome. Neuro Oncol..

[7] Gerstner E.R., Zhang Z., Fink J.R., Muzi M., Hanna L., Greco E., Prah M., Schmainda K.M., Mintz A., Kostakoglu L. (2016). ACRIN 6684: Assessment of Tumor Hypoxia in Newly Diagnosed Glioblastoma Using 18F-FMISO PET and MRI. Clin. Cancer Res..

[8] Triner D., Shah Y.M. (2016). Hypoxia-inducible factors: A central link between inflammation and cancer. J. Clin. Investig..

[9] Anobile D.P., Montenovo G., Pecoraro C., Franczak M., Ait Iddouch W., Peters G.J., Riganti C., Giovannetti E. (2022). Splicing deregulation, microRNA and notch aberrations: Fighting the three-headed dog to overcome drug resistance in malignant mesothelioma. Expert Rev. Clin. Pharmacol..

[10] Zhang H., Cao K., Xiang J., Zhang M., Zhu M., Xi Q. (2023). Hypoxia induces immunosuppression, metastasis and drug resistance in pancreatic cancers. Cancer Lett..

[11] Yu T., Tang B., Sun X. (2017). Development of Inhibitors Targeting Hypoxia-Inducible Factor 1 and 2 for Cancer Therapy. Yonsei Med. J..

[12] Gilbert M.R., Dignam J.J., Armstrong T.S., Wefel J.S., Blumenthal D.T., Vogelbaum M.A., Colman H., Chakravarti A., Pugh S., Won M. (2014). A randomized trial of bevacizumab for newly diagnosed glioblastoma. N. Engl. J. Med..

[13] Domènech M., Hernández A., Plaja A., Martínez-Balibrea E., Balañà C. (2021). Hypoxia: The Cornerstone of Glioblastoma. Int. J. Mol. Sci..

[14] Matthew J.P., Joanne E.M., Patrick M.B., Isabelle B., Tammy C.H., Cynthia D.M., Larissa S., Jennifer M.T., Elie A.A., Sue E.B. (2021). The PRISMA 2020 statement: An updated guideline for reporting systematic reviews. BMJ.

[15] Van den Akker O.R., Peters G.-J.Y., Bakker C.J., Carlsson R., Coles N.A., Corker K.S., Feldman G., Moreau D., Nordström T., Pickering J.S. (2023). Increasing the transparency of systematic reviews: Presenting a generalized registration form. Syst. Rev..

[16] Hashimoto T., Urushihara Y., Murata Y., Fujishima Y., Hosoi Y. (2022). AMPK increases expression of ATM through transcriptional factor Sp1 and induces radioresistance under severe hypoxia in glioblastoma cell lines. Biochem. Biophys. Res. Commun..

[17] Ho K.H., Shih C.M., Liu A.J., Chen K.C. (2022). Hypoxia-inducible lncRNA MIR210HG interacting with OCT1 is involved in glioblastoma multiforme malignancy. Cancer Sci..

[18] Ishikawa T., Ogura Y., Tanaka K., Nagashima H., Sasayama T., Endo M., Minami Y. (2023). Ror1 is expressed inducibly by Notch and hypoxia signaling and regulates stem cell-like property of glioblastoma cells. Cancer Sci..

[19] Agrawal R., Pandey P., Jha P., Dwivedi V., Sarkar C., Kulshreshtha R. (2014). Hypoxic signature of microRNAs in glioblastoma: Insights from small RNA deep sequencing. BMC Genom..

[20] Bianco A.M., Uno M., Oba-Shinjo S.M., Clara C.A., de Almeida Galatro T.F., Rosemberg S., Teixeira M.J., Nagahashi Marie S.K. (2015). CXCR7 and CXCR4 Expressions in Infiltrative Astrocytomas and Their Interactions with HIF1α Expression and IDH1 Mutation. Pathol. Oncol. Res..

[21] Eckerich C., Zapf S., Fillbrandt R., Loges S., Westphal M., Lamszus K. (2007). Hypoxia can induce c-Met expression in glioma cells and enhance SF/HGF-induced cell migration. Int. J. Cancer.

[22] Inukai M., Yokoi A., Ishizuka Y., Hashimura M., Matsumoto T., Oguri Y., Nakagawa M., Ishibashi Y., Ito T., Kumabe T. (2022). A functional role of S100A4/non-muscle myosin IIA axis for pro-tumorigenic vascular functions in glioblastoma. Cell Commun. Signal.

[23] Kimura H., Weisz A., Kurashima Y., Hashimoto K., Ogura T., D’Acquisto F., Addeo R., Makuuchi M., Esumi H. (2000). Hypoxia response element of the human vascular endothelial growth factor gene mediates transcriptional regulation by nitric oxide: Control of hypoxia-inducible factor-1 activity by nitric oxide. Blood.

[24] Li J., Chen Q., Xu S., Wu J., Huang Q., Song P., Duan F. (2018). Down-regulation of BAG3 inhibits proliferation and promotes apoptosis of glioblastoma multiforme through BAG3/HSP70/HIF-1α signaling pathway. Int. J. Clin. Exp. Pathol..

[25] Méndez O., Zavadil J., Esencay M., Lukyanov Y., Santovasi D., Wang S.C., Newcomb E.W., Zagzag D. (2010). Knock down of HIF-1alpha in glioma cells reduces migration in vitro and invasion in vivo and impairs their ability to form tumor spheres. Mol. Cancer.

[26] Miska J., Lee-Chang C., Rashidi A., Muroski M.E., Chang A.L., Lopez-Rosas A., Zhang P., Panek W.K., Cordero A., Han Y. (2019). HIF-1α Is a Metabolic Switch between Glycolytic-Driven Migration and Oxidative Phosphorylation-Driven Immunosuppression of Tregs in Glioblastoma. Cell Rep..

[27] Mohapatra S.R., Sadik A., Tykocinski L.O., Dietze J., Poschet G., Heiland I., Opitz C.A. (2019). Hypoxia Inducible Factor 1α Inhibits the Expression of Immunosuppressive Tryptophan-2,3-Dioxygenase in Glioblastoma. Front. Immunol..

[28] Mongiardi M.P., Savino M., Falchetti M.L., Illi B., Bozzo F., Valle C., Helmer-Citterich M., Ferrè F., Nasi S., Levi A. (2016). c-MYC inhibition impairs hypoxia response in glioblastoma multiforme. Oncotarget.

[29] Nie W., Luo X., Lu D., Yuan P., Liu B., Xu H., Ye M. (2022). Casein kinase 1α 1 is involved in the progression of glioblastoma through HIF-1α-mediated autophagy. J. Neurophysiol..

[30] Noch E., Bookland M., Khalili K. (2011). Astrocyte-elevated gene-1 (AEG-1) induction by hypoxia and glucose deprivation in glioblastoma. Cancer Biol. Ther..

[31] Pistollato F., Rampazzo E., Abbadi S., Della Puppa A., Scienza R., D’Avella D., Denaro L., Te Kronnie G., Panchision D.M., Basso G. (2009). Molecular mechanisms of HIF-1alpha modulation induced by oxygen tension and BMP2 in glioblastoma derived cells. PLoS ONE.

[32] Qiang L., Wu T., Zhang H.W., Lu N., Hu R., Wang Y.J., Zhao L., Chen F.H., Wang X.T., You Q.D. (2012). HIF-1α is critical for hypoxia-mediated maintenance of glioblastoma stem cells by activating Notch signaling pathway. Cell Death Differ..

[33] Said H.M., Polat B., Stein S., Guckenberger M., Hagemann C., Staab A., Katzer A., Anacker J., Flentje M., Vordermark D. (2012). Inhibition of N-Myc down regulated gene 1 in in vitro cultured human glioblastoma cells. World J. Clin. Oncol..

[34] Sesen J., Cammas A., Scotland S.J., Elefterion B., Lemarié A., Millevoi S., Mathew L.K., Seva C., Toulas C., Moyal E.C. (2014). Int6/eIF3e is essential for proliferation and survival of human glioblastoma cells. Int. J. Mol. Sci..

[35] Rong Y., Hu F., Huang R., Mackman N., Horowitz J.M., Jensen R.L., Durden D.L., Van Meir E.G., Brat D.J. (2006). Early growth response gene-1 regulates hypoxia-induced expression of tissue factor in glioblastoma multiforme through hypoxia-inducible factor-1-independent mechanisms. Cancer Res..

[36] Fan X., Fan J., Yang H., Zhao C., Niu W., Fang Z., Chen X. (2021). Heterogeneity of subsets in glioblastoma mediated by Smad3 palmitoylation. Oncogenesis.

[37] Voss D.M., Sloan A., Spina R., Ames H.M., Bar E.E. (2020). The Alternative Splicing Factor, MBNL1, Inhibits Glioblastoma Tumor Initiation and Progression by Reducing Hypoxia-Induced Stemness. Cancer Res..

[38] Wang P., Yan Q., Liao B., Zhao L., Xiong S., Wang J., Zou D., Pan J., Wu L., Deng Y. (2020). The HIF1α/HIF2α-miR210-3p network regulates glioblastoma cell proliferation, dedifferentiation and chemoresistance through EGF under hypoxic conditions. Cell Death Dis..

[39] Bae W.Y., Choi J.S., Nam S., Jeong J.W. (2021). β-arrestin 2 stimulates degradation of HIF-1α and modulates tumor progression of glioblastoma. Cell Death Differ..

[40] Feng J., Zhang Y., She X., Sun Y., Fan L., Ren X., Fu H., Liu C., Li P., Zhao C. (2019). Hypermethylated gene ANKDD1A is a candidate tumor suppressor that interacts with FIH1 and decreases HIF1α stability to inhibit cell autophagy in the glioblastoma multiforme hypoxia microenvironment. Oncogene.

[41] Nishikawa M., Inoue A., Ohnishi T., Yano H., Ozaki S., Kanemura Y., Suehiro S., Ohtsuka Y., Kohno S., Ohue S. (2021). Hypoxia-induced phenotypic transition from highly invasive to less invasive tumors in glioma stem-like cells: Significance of CD44 and osteopontin as therapeutic targets in glioblastoma. Transl. Oncol..

[42] Choksi S., Lin Y., Pobezinskaya Y., Chen L., Park C., Morgan M., Li T., Jitkaew S., Cao X., Kim Y.S. (2011). A HIF-1 target, ATIA, protects cells from apoptosis by modulating the mitochondrial thioredoxin, TRX2. Mol. Cell.

[43] Lee J., Kim E., Chong K., Ryu S.W., Kim C., Choi K., Kim J.H., Choi C. (2022). Atypical induction of HIF-1α expression by pericellular Notch1 signaling suffices for the malignancy of glioblastoma multiforme cells. Cell Mol. Life Sci..

[44] Katakowski M., Charteris N., Chopp M., Khain E. (2016). Density-Dependent Regulation of Glioma Cell Proliferation and Invasion Mediated by miR-9. Cancer Microenviron..

[45] Ji X., Wang H., Zhu J., Zhu L., Pan H., Li W., Zhou Y., Cong Z., Yan F., Chen S. (2014). Knockdown of Nrf2 suppresses glioblastoma angiogenesis by inhibiting hypoxia-induced activation of HIF-1α. Int. J. Cancer.

[46] Gauthier L.R., Saati M., Bensalah-Pigeon H., Ben M’Barek K., Gitton-Quent O., Bertrand R., Busso D., Mouthon M.A., Collura A., Junier M.P. (2020). The HIF1α/JMY pathway promotes glioblastoma stem-like cell invasiveness after irradiation. Sci. Rep..

[47] Hu Q., Liu F., Yan T., Wu M., Ye M., Shi G., Lv S., Zhu X. (2019). MicroRNA-576-3p inhibits the migration and proangiogenic abilities of hypoxia-treated glioma cells through hypoxia-inducible factor-1α. Int. J. Mol. Med..

[48] Ghosh S., Paul A., Sen E. (2013). Tumor necrosis factor α-induced hypoxia-inducible factor 1α-β-catenin axis regulates major histocompatibility complex class I gene activation through chromatin remodeling. Mol. Cell Biol..

[49] Evagelou S.L., Bebenek O., Specker E.J., Uniacke J. (2020). DEAD Box Protein Family Member DDX28 Is a Negative Regulator of Hypoxia-Inducible Factor 2α- and Eukaryotic Initiation Factor 4E2-Directed Hypoxic Translation. Mol. Cell Biol..

[50] Ikemori R.Y., Machado C.M., Furuzawa K.M., Nonogaki S., Osinaga E., Umezawa K., de Carvalho M.A., Verinaud L., Chammas R. (2014). Galectin-3 up-regulation in hypoxic and nutrient deprived microenvironments promotes cell survival. PLoS ONE.

[51] Man J., Yu X., Huang H., Zhou W., Xiang C., Huang H., Miele L., Liu Z., Bebek G., Bao S. (2018). Hypoxic Induction of Vasorin Regulates Notch1 Turnover to Maintain Glioma Stem-like Cells. Cell Stem Cell.

[52] Bordji K., Grandval A., Cuhna-Alves L., Lechapt-Zalcman E., Bernaudin M. (2014). Hypoxia-inducible factor-2α (HIF-2α), but not HIF-1α, is essential for hypoxic induction of class III β-tubulin expression in human glioblastoma cells. FEBS J..

[53] Maurer G.D., Heller S., Wanka C., Rieger J., Steinbach J.P. (2019). Knockdown of the TP53-Induced Glycolysis and Apoptosis Regulator (TIGAR) Sensitizes Glioma Cells to Hypoxia, Irradiation and Temozolomide. Int. J. Mol. Sci..

[54] Fan Y., Potdar A.A., Gong Y., Eswarappa S.M., Donnola S., Lathia J.D., Hambardzumyan D., Rich J.N., Fox P.L. (2014). Profilin-1 phosphorylation directs angiocrine expression and glioblastoma progression through HIF-1α accumulation. Nat. Cell Biol..

[55] Wei J., Zhu K., Yang Z., Zhou Y., Xia Z., Ren J., Zhao Y., Wu G., Liu C. (2023). Hypoxia-Induced Autophagy Is Involved in Radioresistance via HIF1A-Associated Beclin-1 in Glioblastoma Multiforme. Heliyon.

[56] Coma S., Shimizu A., Klagsbrun M. (2011). Hypoxia induces tumor and endothelial cell migration in a semaphorin 3F- and VEGF-dependent manner via transcriptional repression of their common receptor neuropilin 2. Cell Adhes. Migr..

[57] Bao L., Chen Y., Lai H.T., Wu S.Y., Wang J.E., Hatanpaa K.J., Raisanen J.M., Fontenot M., Lega B., Chiang C.M. (2018). Methylation of hypoxia-inducible factor (HIF)-1α by G9a/GLP inhibits HIF-1 transcriptional activity and cell migration. Nucleic Acids Res..

[58] Lim K.S., Lim K.J., Price A.C., Orr B.A., Eberhart C.G., Bar E.E. (2014). Inhibition of monocarboxylate transporter-4 depletes stem-like glioblastoma cells and inhibits HIF transcriptional response in a lactate-independent manner. Oncogene.

[59] Lei F.J., Chiang J.Y., Chang H.J., Chen D.C., Wang H.L., Yang H.A., Wei K.Y., Huang Y.C., Wang C.C., Wei S.T. (2023). Cellular and exosomal GPx1 are essential for controlling hydrogen peroxide balance and alleviating oxidative stress in hypoxic glioblastoma. Redox Biol..

[60] Joshi S., Singh A.R., Durden D.L. (2014). MDM2 regulates hypoxic hypoxia-inducible factor 1α stability in an E3 ligase, proteasome, and PTEN-phosphatidylinositol 3-kinase-AKT-dependent manner. J. Biol. Chem..

[61] Lulli V., Buccarelli M., Ilari R., Castellani G., De Dominicis C., Di Giamberardino A., QG D.A., Giannetti S., Martini M., Stumpo V. (2020). Mir-370-3p Impairs Glioblastoma Stem-Like Cell Malignancy Regulating a Complex Interplay between HMGA2/HIF1A and the Oncogenic Long Non-Coding RNA (lncRNA) NEAT1. Int. J. Mol. Sci..

[62] Jung J., Zhang Y., Celiku O., Zhang W., Song H., Williams B.J., Giles A.J., Rich J.N., Abounader R., Gilbert M.R. (2019). Mitochondrial NIX Promotes Tumor Survival in the Hypoxic Niche of Glioblastoma. Cancer Res..

[63] Jin X., Kuang Y., Li L., Li H., Zhao T., He Y., Di C., Kang J., Yuan L., Yu B. (2022). A positive feedback circuit comprising p21 and HIF-1α aggravates hypoxia-induced radioresistance of glioblastoma by promoting Glut1/LDHA-mediated glycolysis. FASEB J..

[64] Nardinocchi L., Pantisano V., Puca R., Porru M., Aiello A., Grasselli A., Leonetti C., Safran M., Rechavi G., Givol D. (2010). Zinc downregulates HIF-1α and inhibits its activity in tumor cells in vitro and in vivo. PLoS ONE.

[65] Maugeri G., D’Amico A.G., Saccone S., Federico C., Rasà D.M., Caltabiano R., Broggi G., Giunta S., Musumeci G., D’Agata V. (2021). Effect of PACAP on Hypoxia-Induced Angiogenesis and Epithelial-Mesenchymal Transition in Glioblastoma. Biomedicines.

[66] Ma S., Wang F., Dong J., Wang N., Tao S., Du J., Hu S. (2022). Inhibition of hypoxia-inducible factor 1 by acriflavine renders glioblastoma sensitive for photodynamic therapy. J. Photochem. Photobiol. B.

[67] D’Amico A.G., Maugeri G., Magrì B., Giunta S., Saccone S., Federico C., Pricoco E., Broggi G., Caltabiano R., Musumeci G. (2023). Modulatory activity of ADNP on the hypoxia-induced angiogenic process in glioblastoma. Int. J. Oncol..

[68] D’Alessio A., Proietti G., Lama G., Biamonte F., Lauriola L., Moscato U., Vescovi A., Mangiola A., Angelucci C., Sica G. (2016). Analysis of angiogenesis related factors in glioblastoma, peritumoral tissue and their derived cancer stem cells. Oncotarget.

[69] Cristofaro I., Limongi C., Piscopo P., Crestini A., Guerriero C., Fiore M., Conti L., Confaloni A., Tata A.M. (2020). M2 Receptor Activation Counteracts the Glioblastoma Cancer Stem Cell Response to Hypoxia Condition. Int. J. Mol. Sci..

[70] Gagner J.P., Sarfraz Y., Ortenzi V., Alotaibi F.M., Chiriboga L.A., Tayyib A.T., Douglas G.J., Chevalier E., Romagnoli B., Tuffin G. (2017). Multifaceted C-X-C Chemokine Receptor 4 (CXCR4) Inhibition Interferes with Anti-Vascular Endothelial Growth Factor Therapy-Induced Glioma Dissemination. Am. J. Pathol..

[71] Lin L., Luo J., Wang Z., Cai X. (2024). Borneol promotes autophagic degradation of HIF-1α and enhances chemotherapy sensitivity in malignant glioma. PeerJ.

[72] Douglas C., Lomeli N., Lepe J., Di K., Nandwana N.K., Vu T., Pham J., Kenney M.C., Das B., Bota D.A. (2023). Discovery and Validation of Novel LonP1 and Proteasome Inhibitor in IDH1-R132H Malignant Astrocytoma Models. bioRxiv.

[73] Arienti C., Pignatta S., Zanoni M., Zamagni A., Cortesi M., Sarnelli A., Romeo A., Arpa D., Longobardi P., Bartolini D. (2021). High-pressure oxygen rewires glucose metabolism of patient-derived glioblastoma cells and fuels inflammasome response. Cancer Lett..

[74] Lin K.W., Liao A., Qutub A.A. (2015). Simulation predicts IGFBP2-HIF1α interaction drives glioblastoma growth. PLoS Comput. Biol..

[75] Lund E.L., Høg A., Olsen M.W., Hansen L.T., Engelholm S.A., Kristjansen P.E. (2004). Differential regulation of VEGF, HIF1alpha and angiopoietin-1, -2 and -4 by hypoxia and ionizing radiation in human glioblastoma. Int. J. Cancer.

[76] Hofstetter C.P., Burkhardt J.K., Shin B.J., Gürsel D.B., Mubita L., Gorrepati R., Brennan C., Holland E.C., Boockvar J.A. (2012). Protein phosphatase 2A mediates dormancy of glioblastoma multiforme-derived tumor stem-like cells during hypoxia. PLoS ONE.

[77] Bi L., Liu Y., Yang Q., Zhou X., Li H., Liu Y., Li J., Lu Y., Tang H. (2021). Paris saponin H inhibits the proliferation of glioma cells through the A1 and A3 adenosine receptor-mediated pathway. Int. J. Mol. Med..

[78] Khoei S., Shoja M., Mostaar A., Faeghi F. (2016). Effects of resveratrol and methoxyamine on the radiosensitivity of iododeoxyuridine in U87MG glioblastoma cell line. Exp. Biol. Med..

[79] Liu F., Xu X., Li C., Li C., Li Y., Yin S., Yu S., Chen X.Q. (2020). Mannose synergizes with chemoradiotherapy to cure cancer via metabolically targeting HIF-1 in a novel triple-negative glioblastoma mouse model. Clin. Transl. Med..

[80] Dačević M., Isaković A., Podolski-Renić A., Isaković A.M., Stanković T., Milošević Z., Rakić L., Ruždijić S., Pešić M. (2013). Purine nucleoside analog--sulfinosine modulates diverse mechanisms of cancer progression in multi-drug resistant cancer cell lines. PLoS ONE.

[81] Ishii A., Kimura T., Sadahiro H., Kawano H., Takubo K., Suzuki M., Ikeda E. (2016). Histological Characterization of the Tumorigenic “Peri-Necrotic Niche” Harboring Quiescent Stem-Like Tumor Cells in Glioblastoma. PLoS ONE.

[82] Li Y., Wang D., Zhang Z., Wang Y., Zhang Z., Liang Z., Liu F., Chen L. (2023). Photodynamic therapy enhances the cytotoxicity of temozolomide against glioblastoma via reprogramming anaerobic glycolysis. Photodiagn. Photodyn. Ther..

[83] Bernstock J.D., Ye D., Gessler F.A., Lee Y.J., Peruzzotti-Jametti L., Baumgarten P., Johnson K.R., Maric D., Yang W., Kögel D. (2017). Topotecan is a potent inhibitor of SUMOylation in glioblastoma multiforme and alters both cellular replication and metabolic programming. Sci. Rep..

[84] Tafani M., Di Vito M., Frati A., Pellegrini L., De Santis E., Sette G., Eramo A., Sale P., Mari E., Santoro A. (2011). Pro-inflammatory gene expression in solid glioblastoma microenvironment and in hypoxic stem cells from human glioblastoma. J. Neuroinflamm..

[85] Muh C.R., Joshi S., Singh A.R., Kesari S., Durden D.L., Makale M.T. (2014). PTEN status mediates 2ME2 anti-tumor efficacy in preclinical glioblastoma models: Role of HIF1α suppression. J. Neurooncol..

[86] Pore N., Gupta A.K., Cerniglia G.J., Maity A. (2006). HIV protease inhibitors decrease VEGF/HIF-1alpha expression and angiogenesis in glioblastoma cells. Neoplasia.

[87] Sugimoto N., Ishibashi H., Nakamura H., Yachie A., Ohno-Shosaku T. (2017). Hypoxia-induced inhibition of the endocannabinoid system in glioblastoma cells. Oncol. Rep..

[88] Lin C., Lai S.W., Shen C.K., Chen C.W., Tsai C.F., Liu Y.S., Lu D.Y., Huang B.R. (2021). Fenofibrate inhibits hypoxia-inducible factor-1 alpha and carbonic anhydrase expression through activation of AMP-activated protein kinase/HO-1/Sirt1 pathway in glioblastoma cells. Environ. Toxicol..

[89] Gagner J.P., Lechpammer M., Zagzag D. (2018). Induction and Assessment of Hypoxia in Glioblastoma Cells In Vitro. Methods Mol. Biol..

[90] Huang W., Ding X., Ye H., Wang J., Shao J., Huang T. (2018). Hypoxia enhances the migration and invasion of human glioblastoma U87 cells through PI3K/Akt/mTOR/HIF-1α pathway. Neuroreport.

[91] Chhipa R.R., Fan Q., Anderson J., Muraleedharan R., Huang Y., Ciraolo G., Chen X., Waclaw R., Chow L.M., Khuchua Z. (2018). AMP kinase promotes glioblastoma bioenergetics and tumour growth. Nat. Cell Biol..

[92] Pang L., Guo S., Khan F., Dunterman M., Ali H., Liu Y., Huang Y., Chen P. (2023). Hypoxia-driven protease legumain promotes immunosuppression in glioblastoma. Cell Rep. Med..

[93] Hu Y.L., DeLay M., Jahangiri A., Molinaro A.M., Rose S.D., Carbonell W.S., Aghi M.K. (2012). Hypoxia-induced autophagy promotes tumor cell survival and adaptation to antiangiogenic treatment in glioblastoma. Cancer Res..

[94] Chou C.W., Wang C.C., Wu C.P., Lin Y.J., Lee Y.C., Cheng Y.W., Hsieh C.H. (2012). Tumor cycling hypoxia induces chemoresistance in glioblastoma multiforme by upregulating the expression and function of ABCB1. Neuro Oncol..

[95] Barliya T., Mandel M., Livnat T., Weinberger D., Lavie G. (2011). Degradation of HIF-1alpha under hypoxia combined with induction of Hsp90 polyubiquitination in cancer cells by hypericin: A unique cancer therapy. PLoS ONE.

[96] Hsieh C.H., Shyu W.C., Chiang C.Y., Kuo J.W., Shen W.C., Liu R.S. (2011). NADPH oxidase subunit 4-mediated reactive oxygen species contribute to cycling hypoxia-promoted tumor progression in glioblastoma multiforme. PLoS ONE.

[97] Kannappan V., Liu Y., Wang Z., Azar K., Kurusamy S., Kilari R.S., Armesilla A.L., Morris M.R., Najlah M., Liu P. (2022). PLGA-Nano-Encapsulated Disulfiram Inhibits Hypoxia-Induced NF-κB, Cancer Stem Cells, and Targets Glioblastoma In Vitro and In Vivo. Mol. Cancer Ther..

[98] Joseph J.V., Conroy S., Pavlov K., Sontakke P., Tomar T., Eggens-Meijer E., Balasubramaniyan V., Wagemakers M., den Dunnen W.F., Kruyt F.A. (2015). Hypoxia enhances migration and invasion in glioblastoma by promoting a mesenchymal shift mediated by the HIF1α-ZEB1 axis. Cancer Lett..

[99] Caragher S.P., Shireman J.M., Huang M., Miska J., Atashi F., Baisiwala S., Hong Park C., Saathoff M.R., Warnke L., Xiao T. (2019). Activation of Dopamine Receptor 2 Prompts Transcriptomic and Metabolic Plasticity in Glioblastoma. J. Neurosci..

[100] Peng G., Wang Y., Ge P., Bailey C., Zhang P., Zhang D., Meng Z., Qi C., Chen Q., Chen J. (2021). The HIF1α-PDGFD-PDGFRα axis controls glioblastoma growth at normoxia/mild-hypoxia and confers sensitivity to targeted therapy by echinomycin. J. Exp. Clin. Cancer Res..

[101] Han D., Wei W., Chen X., Zhang Y., Wang Y., Zhang J., Wang X., Yu T., Hu Q., Liu N. (2015). NF-κB/RelA-PKM2 mediates inhibition of glycolysis by fenofibrate in glioblastoma cells. Oncotarget.

[102] Dominguez C.L., Floyd D.H., Xiao A., Mullins G.R., Kefas B.A., Xin W., Yacur M.N., Abounader R., Lee J.K., Wilson G.M. (2013). Diacylglycerol kinase α is a critical signaling node and novel therapeutic target in glioblastoma and other cancers. Cancer Discov..

[103] Hsieh C.H., Lin Y.J., Wu C.P., Lee H.T., Shyu W.C., Wang C.C. (2015). Livin contributes to tumor hypoxia-induced resistance to cytotoxic therapies in glioblastoma multiforme. Clin. Cancer Res..

[104] Ahmed E.M., Bandopadhyay G., Coyle B., Grabowska A. (2018). A HIF-independent, CD133-mediated mechanism of cisplatin resistance in glioblastoma cells. Cell. Oncol..

[105] Lee D.H., Cheul Oh S., Giles A.J., Jung J., Gilbert M.R., Park D.M. (2017). Cardiac glycosides suppress the maintenance of stemness and malignancy via inhibiting HIF-1α in human glioma stem cells. Oncotarget.

[106] Bar E.E., Lin A., Mahairaki V., Matsui W., Eberhart C.G. (2010). Hypoxia increases the expression of stem-cell markers and promotes clonogenicity in glioblastoma neurospheres. Am. J. Pathol..

[107] Chen W.L., Wang C.C., Lin Y.J., Wu C.P., Hsieh C.H. (2015). Cycling hypoxia induces chemoresistance through the activation of reactive oxygen species-mediated B-cell lymphoma extra-long pathway in glioblastoma multiforme. J. Transl. Med..

[108] Li L.C., Zhang M., Feng Y.K., Wang X.J. (2020). IDH1-R132H Suppresses Glioblastoma Malignancy through FAT1-ROS-HIF-1α Signaling. Neurol. India.

[109] Ge X., Pan M.H., Wang L., Li W., Jiang C., He J., Abouzid K., Liu L.Z., Shi Z., Jiang B.H. (2018). Hypoxia-mediated mitochondria apoptosis inhibition induces temozolomide treatment resistance through miR-26a/Bad/Bax axis. Cell Death Dis..

[110] Liao Y., Luo Z., Lin Y., Chen H., Chen T., Xu L., Orgurek S., Berry K., Dzieciatkowska M., Reisz J.A. (2022). PRMT3 drives glioblastoma progression by enhancing HIF1A and glycolytic metabolism. Cell Death Dis..

[111] Kioi M., Vogel H., Schultz G., Hoffman R.M., Harsh G.R., Brown J.M. (2010). Inhibition of vasculogenesis, but not angiogenesis, prevents the recurrence of glioblastoma after irradiation in mice. J. Clin. Investig..

[112] Boso D., Rampazzo E., Zanon C., Bresolin S., Maule F., Porcù E., Cani A., Della Puppa A., Trentin L., Basso G. (2019). HIF-1α/Wnt signaling-dependent control of gene transcription regulates neuronal differentiation of glioblastoma stem cells. Theranostics.

[113] Hu J., Wang X.F. (2016). HIF-miR-215-KDM1B promotes glioma-initiating cell adaptation to hypoxia. Cell Cycle.

[114] Chen W., Cheng X., Wang X., Wang J., Wen X., Xie C., Liao C. (2019). Clinical implications of hypoxia-inducible factor-1α and caveolin-1 overexpression in isocitrate dehydrogenase-wild type glioblastoma multiforme. Oncol. Lett..

[115] Bache M., Rot S., Keßler J., Güttler A., Wichmann H., Greither T., Wach S., Taubert H., Söling A., Bilkenroth U. (2015). mRNA expression levels of hypoxia-induced and stem cell-associated genes in human glioblastoma. Oncol. Rep..

[116] Erpolat O.P., Gocun P.U., Akmansu M., Ozgun G., Akyol G. (2013). Hypoxia-related molecules HIF-1α, CA9, and osteopontin: Predictors of survival in patients with high-grade glioma. Strahlenther. Onkol..

[117] Kaynar M.Y., Sanus G.Z., Hnimoglu H., Kacira T., Kemerdere R., Atukeren P., Gumustas K., Canbaz B., Tanriverdi T. (2008). Expression of hypoxia inducible factor-1alpha in tumors of patients with glioblastoma multiforme and transitional meningioma. J. Clin. Neurosci..

[118] Irie N., Matsuo T., Nagata I. (2004). Protocol of radiotherapy for glioblastoma according to the expression of HIF-1. Brain Tumor Pathol..

[119] Ji X., Wang H., Zhu J., Tang Y., Zhou Y., Zhu L., Gao C., Li W., You W., Yu B. (2013). Correlation of Nrf2 and HIF-1α in glioblastoma and their relationships to clinicopathologic features and survival. Neurol. Res..

[120] Sfifou F., Hakkou E.M., Bouaiti E.A., Slaoui M., Errihani H., Al Bouzidi A., Abouqal R., El Ouahabi A., Cherradi N. (2021). Correlation of immunohistochemical expression of HIF-1alpha and IDH1 with clinicopathological and therapeutic data of moroccan glioblastoma and survival analysis. Ann. Med. Surg..

[121] Potharaju M., Mathavan A., Mangaleswaran B., Patil S., John R., Ghosh S., Kalavakonda C., Ghosh M., Verma R.S. (2019). Clinicopathological Analysis of HIF-1alpha and TERT on Survival Outcome in Glioblastoma Patients: A Prospective, Single Institution Study. J. Cancer.

[122] Clara C.A., Marie S.K., de Almeida J.R., Wakamatsu A., Oba-Shinjo S.M., Uno M., Neville M., Rosemberg S. (2014). Angiogenesis and expression of PDGF-C, VEGF, CD105 and HIF-1α in human glioblastoma. Neuropathology.

[123] El-Benhawy S.A., Sakr O.A., Fahmy E.I., Ali R.A., Hussein M.S., Nassar E.M., Salem S.M., Abu-Samra N., Elzawawy S. (2022). Assessment of Serum Hypoxia Biomarkers Pre- and Post-radiotherapy in Patients with Brain Tumors. J. Mol. Neurosci..

[124] Begagić E., Pugonja R., Bečulić H., Čeliković A., Tandir Lihić L., Kadić Vukas S., Čejvan L., Skomorac R., Selimović E., Jaganjac B. (2023). Molecular Targeted Therapies in Glioblastoma Multiforme: A Systematic Overview of Global Trends and Findings. Brain Sci..

[125] Zhang H.Y., Yu H.Y., Zhao G.X., Jiang X.Z., Gao G., Wei B.J. (2023). Global research trends in immunotherapy for glioma: A comprehensive visualization and bibliometric analysis. Front. Endocrinol..

[126] Łaba A.E., Ziółkowski P. (2021). Trends in glioblastoma treatment research: An analysis of clinical trials and literature. Neurol. Neurochir. Pol..

[127] Suzuki K., Gerelchuluun A., Hong Z., Sun L., Zenkoh J., Moritake T., Tsuboi K. (2013). Celecoxib enhances radiosensitivity of hypoxic glioblastoma cells through endoplasmic reticulum stress. Neuro Oncol..

[128] Barzegar Behrooz A., Talaie Z., Jusheghani F., Łos M.J., Klonisch T., Ghavami S. (2022). Wnt and PI3K/Akt/mTOR Survival Pathways as Therapeutic Targets in Glioblastoma. Int. J. Mol. Sci..

[129] Ross A.C. (2012). Use of laboratory studies for the design, explanation, and validation of human micronutrient intervention studies. J. Nutr..

[130] Ortega M.A., Fraile-Martinez O., García-Montero C., Callejón-Peláez E., Sáez M.A., Álvarez-Mon M.A., García-Honduvilla N., Monserrat J., Álvarez-Mon M., Bujan J. (2021). A General Overview on the Hyperbaric Oxygen Therapy: Applications, Mechanisms and Translational Opportunities. Medicina.

[131] Chen S.-Y., Tsuneyama K., Yen M.-H., Lee J.-T., Chen J.-L., Huang S.-M. (2021). Hyperbaric oxygen suppressed tumor progression through the improvement of tumor hypoxia and induction of tumor apoptosis in A549-cell-transferred lung cancer. Sci. Rep..

[132] Herrera-Campos A.B., Zamudio-Martinez E., Delgado-Bellido D., Fernández-Cortés M., Montuenga L.M., Oliver F.J., Garcia-Diaz A. (2022). Implications of Hyperoxia over the Tumor Microenvironment: An Overview Highlighting the Importance of the Immune System. Cancers.

[133] Trejo-Solis C., Silva-Adaya D., Serrano-García N., Magaña-Maldonado R., Jimenez-Farfan D., Ferreira-Guerrero E., Cruz-Salgado A., Castillo-Rodriguez R.A. (2023). Role of Glycolytic and Glutamine Metabolism Reprogramming on the Proliferation, Invasion, and Apoptosis Resistance through Modulation of Signaling Pathways in Glioblastoma. Int. J. Mol. Sci..

[134] Beylerli O., Beilerli A., Shumadalova A., Wang X., Yang M., Sun H., Teng L. (2022). Therapeutic effect of natural polyphenols against glioblastoma. Front. Cell Dev. Biol..

[135] Luís Â., Amaral L., Domingues F., Pereira L., Cascalheira J.F. (2024). Action of Curcumin on Glioblastoma Growth: A Systematic Review with Meta-Analysis of Animal Model Studies. Biomedicines.

[136] Hagiwara F., Omata D., Munakata L., Kageyama S., Maruyama K., Kudo N., Suzuki R. (2023). Brain Delivery of Cisplatin Using Microbubbles in Combination with Ultrasound as an Effective Therapy for Glioblastoma. Pharmaceuticals.

[137] Chédeville A.L., Madureira P.A. (2021). The Role of Hypoxia in Glioblastoma Radiotherapy Resistance. Cancers.

[138] Muniraj N., Siddharth S., Sharma D. (2019). Bioactive Compounds: Multi-Targeting Silver Bullets for Preventing and Treating Breast Cancer. Cancers.

[139] Di Nunzio M.R., Douhal A. (2023). Robust Inclusion Complex of Topotecan Comprised within a Rhodamine-Labeled β-Cyclodextrin: Competing Proton and Energy Transfer Processes. Pharmaceutics.

[140] Ohnishi T. (2024). Current Status and Future Perspective in Glioma Invasion Research. Brain Sci..

[141] Bui B.P., Nguyen P.L., Lee K., Cho J. (2022). Hypoxia-Inducible Factor-1: A Novel Therapeutic Target for the Management of Cancer, Drug Resistance, and Cancer-Related Pain. Cancers.

[142] Bartoszewska S., Sławski J., Collawn J.F., Bartoszewski R. (2023). HIF-1-Induced hsa-miR-429: Understanding Its Direct Targets as the Key to Developing Cancer Diagnostics and Therapies. Cancers.

[143] Englert-Golon M., Tokłowicz M., Żbikowska A., Sajdak S., Kotwicka M., Andrusiewicz M. (2022). Differential Expression of HIF1A, EPAS1, and VEGF Genes in Benign and Malignant Ovarian Neoplasia. Cancers.

[144] Li Z., Bao S., Wu Q., Wang H., Eyler C., Sathornsumetee S., Shi Q., Cao Y., Lathia J., McLendon R.E. (2009). Hypoxia-inducible factors regulate tumorigenic capacity of glioma stem cells. Cancer Cell.

[145] Ramar V., Guo S., Hudson B., Liu M. (2024). Progress in Glioma Stem Cell Research. Cancers.

[146] Monteiro A.R., Hill R., Pilkington G.J., Madureira P.A. (2017). The Role of Hypoxia in Glioblastoma Invasion. Cells.

[147] Oishi T., Koizumi S., Kurozumi K. (2022). Molecular Mechanisms and Clinical Challenges of Glioma Invasion. Brain Sci..

[148] Qannita R.A., Alalami A.I., Harb A.A., Aleidi S.M., Taneera J., Abu-Gharbieh E., El-Huneidi W., Saleh M.A., Alzoubi K.H., Semreen M.H. (2024). Targeting Hypoxia-Inducible Factor-1 (HIF-1) in Cancer: Emerging Therapeutic Strategies and Pathway Regulation. Pharmaceuticals.

[149] Park J.H., Lee H.K. (2022). Current Understanding of Hypoxia in Glioblastoma Multiforme and Its Response to Immunotherapy. Cancers.

[150] Pantazopoulou V., Jeannot P., Rosberg R., Berg T.J., Pietras A. (2021). Hypoxia-Induced Reactivity of Tumor-Associated Astrocytes Affects Glioma Cell Properties. Cells.

[151] Obrador E., Moreno-Murciano P., Oriol-Caballo M., López-Blanch R., Pineda B., Gutiérrez-Arroyo J.L., Loras A., Gonzalez-Bonet L.G., Martinez-Cadenas C., Estrela J.M. (2024). Glioblastoma Therapy: Past, Present and Future. Int. J. Mol. Sci..

